# *In vitro* effects of phytochemicals on adipogenesis with a focus on molecular mechanisms: A systematic review

**DOI:** 10.22038/ijbms.2025.78924.17090

**Published:** 2025

**Authors:** Niusha Kazemi, Elham Ramazani, Zahra Tayarani-Najaran

**Affiliations:** 1 Medical Toxicology Research Center, Mashhad University of Medical Sciences, Mashhad, Iran; 2 Department of Biology, Yazd University, Yazd, Iran; 3 Targeted Drug Delivery Research Center, Pharmaceutical Technology Institute, Mashhad University of Medical Sciences, Mashhad, Iran

**Keywords:** Adipogenesism, In vitro models, Molecular mechanism, Obesity, Phytochemicals, Systematic review

## Abstract

Adipogenesis, the process of proliferation of adipocyte progenitor cells and their differentiation into mature adipocytes, plays a critical role in the development of obesity. In this context, exploring the effects of phytochemicals on adipogenesis is very promising, as nowadays, they are widely used as food, drink, or supplement and can significantly impact general health and obesity control. This systematic review attempts to evaluate new findings regarding the molecular mechanisms of different phytochemicals on adipogenesis in *in vitro *models. Between 2010 and July 2023, a comprehensive systematic search of PubMed and Scopus databases was conducted. The following keywords were used: (“adipogenic”) AND (“inhibit” OR “suppress” OR “reduce” OR “anti” OR “decrease”) AND (“cell” OR “cell line” OR “adipocyte”) AND (“phytochemical” OR “plant” OR “herb”). In this review, 109 studies were comprehensively analyzed, which provided important insights into the process of adipogenesis. Among the numerous transcription factors studied, PPARγ, C/EBPα, and SREBP1c were found to be the most important regulators actively involved in adipocyte differentiation. These results highlight the critical role of these factors in the control of adipogenesis and suggest that they represent promising targets for therapeutic interventions aimed at reducing the excessive lipid accumulation associated with obesity. This study provides a compelling rationale for further exploring phytochemicals as potential therapeutics for treating obesity. The potential benefits of using natural products to influence adipogenesis are evident, and future studies should focus on translating these findings into clinical applications.

## Introduction

Obesity results from an increase in the number and size of fat cells. Increased adipocytes can trigger other chronic diseases such as type 2 diabetes, cardiovascular disease, hypertension, coronary heart disease, atherosclerosis, and some types of cancer. Therefore, understanding the mechanisms of adipogenesis may be helpful to treat obesity and its associated metabolic disorders (1-3). High-cost synthetic drugs for obesity treatment, such as orlistat, lorcaserin, naltrexone, and liraglutide, have shown successful weight loss compared with placebo (4). However, several adverse effects have been reported for them, including cardiovascular toxic effects, hallucinations, headaches, anxiety, and severe hepatic side effects. To combat these problems, studies have been conducted on the anti-obesity effects of various natural bioactive compounds. These bioactive components reduce adipose tissue mass by promoting lipolysis, inhibiting adipose tissue development and preadipocyte differentiation (5). As a result, natural products attract much attention in treating obesity and diabetes, as they have fewer side effects (6). Phytochemicals, compounds produced by plants, have been shown to be potential inhibitors of adipogenesis and may be a promising approach to prevent and treat chronic diseases associated with obesity. Phytochemicals regulate metabolic pathways involved in lipid absorption, energy uptake, burning, lipolysis, and adipocyte differentiation and proliferation, thereby exerting their effects against obesity (7, 8). In addition, phytochemicals have potential therapeutic benefits due to their anti-inflammatory and anti-oxidant properties (9, 10). Examples of secondary plant compounds with anti-adipogenic effects are polyphenols such as resveratrol and quercetin and flavonoids such as apigenin and naringenin (11).

**Figure 1:**
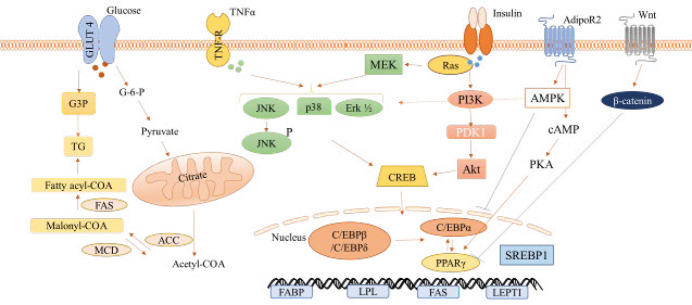
Preadipocyte to adipocyte differentiation

Therefore, the effect of phytochemicals on adipogenesis has been the subject of many studies. Evidence showed that adipocyte differentiation during adipogenesis altered cell phenotype, hormone homeostasis, and transcription factor expression. Based on the results from numerous studies, it was reported that during adipocyte differentiation, several transcription factors are expressed that stimulate adipogenesis. These factors include peroxisome proliferator-activated receptor γ (PPARγ) and CCAAT/enhancer-binding protein α (C/EBPα), which activate adipogenesis (12). On the other hand, the inhibitory effects of Wnt/βcatenin and AMPK pathways on adipocyte differentiation have been reported in some studies (13, 14) (Figure 1). 

Given the characteristics of different phytochemicals in the adipogenesis process, it is assumed that phytochemicals may differ in molecular targets and mechanisms of action. This study aimed to evaluate the main molecular pathways which phytochemicals could alter in *in vitro* models. This systematic review discusses selected phytochemicals and herbs and their effects on adipogenesis, lipogenesis, lipolysis, oxidation of fatty acids, and browning in *in vitro *models. It achieves reliable insight into phytochemicals’ effects on adipogenesis, and additional clinical and animal studies are required. 

## Materials and Methods

The checklist and flowchart of the PRISMA (Preferred Reporting for Systematic Reviews and Meta-Analyses) guidelines were followed for this systematic review.

### Search strategy

Two online databases, PubMed and Scopus, were searched for articles published between 2010 and July 2023. The complete electronic search was conducted using the following keywords and combinations: ”adipogenic” AND “inhibit” OR “suppress” OR “reduce” OR “anti” OR “decrease”) AND (“cell” OR “cell line” OR “adipocyte”) AND (“phytochemical” OR “plant” OR “herb”). There was no restriction on language during the search and identification of studies. The search strategy was re-run to retrieve the most recent studies eligible for inclusion.

### Study selection/ inclusion and exclusion criteria

To investigate the *in vitro* protective effects of phytochemicals on adipogenesis, we performed a careful literature search in scientific databases. The criteria for establishing study eligibility were defined in terms of participants**, **intervention, comparison, outcomes**, **and study design (Table 1). Specific inclusion criteria for *in vitro* studies included the following: (a) published in English; (b) administered the effects of plants with different groups of phytochemicals; (c) *in vitro* experiments on adipogenesis and lipid accumulation; and (d) published in peer-reviewed journals. Additionally, irrelevant documents, reviews or letters, duplicates, editorials, incomplete articles, conference papers, book chapters, and experiments without testing the efficacy of phytochemicals on *in vitro* models were excluded. The articles were evaluated in the following order: first, the titles and abstracts were screened for eligibility, and then the articles that appeared to meet the inclusion criteria were collected.

### Data extraction

The three authors (Niusha Kazemi, Elham Ramazani, and Zahra Tayarani-Najaran) extracted relevant information from the included articles. The information includes the first author and year, country, cell line, intervention, concentration/duration, and intervention results (outcomes).

### Adipogenesis, lipid accumulation, and fat content assessments

Differentiation medium (DM), DMEM (Dulbecco’s Modified Eagle Medium), and MDI (insulin, dexamethasone, and isobutylmethylxanthine) were used to differentiate preadipocytes into adipocytes (13, 15). Intracellular lipids and triglyceride (TG) were determined by oil red O staining (16). In parallel, reverse transcription polymerase chain reaction (RT-PCR) was used to determine the concentration of transcription factors (17, 18). The statistically significant elevation in adipogenesis was considered a successful model for measuring lipid accumulation and tracking the associated metabolic pathways.

### Outcome measures

*Primary endpoint*

The authors considered decreased adipogenesis and lipid accumulation as a primary outcome. These endpoints were evaluated by changes in the formation of lipid droplets after differentiation of preadipocytes measuring by oil red O staining agent.

*Secondary endpoint*

The authors examined changes in key regulators such as PPARγ, C/EBPα, SREBP-1c, and the AMPK signaling pathway after adipocyte differentiation compared to control groups. The secondary outcomes were measured at the end of the differentiating process. In some cases, we also checked cell cycle modifications relevant to our results.

### Risk of bias in individual studies

The QUIN tool for *in vitro* studies was used to assess the risk of bias in individual studies. A summary evaluation was also provided. First, the authors independently assessed the data and then resolved disagreements (19).

## Results

### Study selection

**Figure 2:**
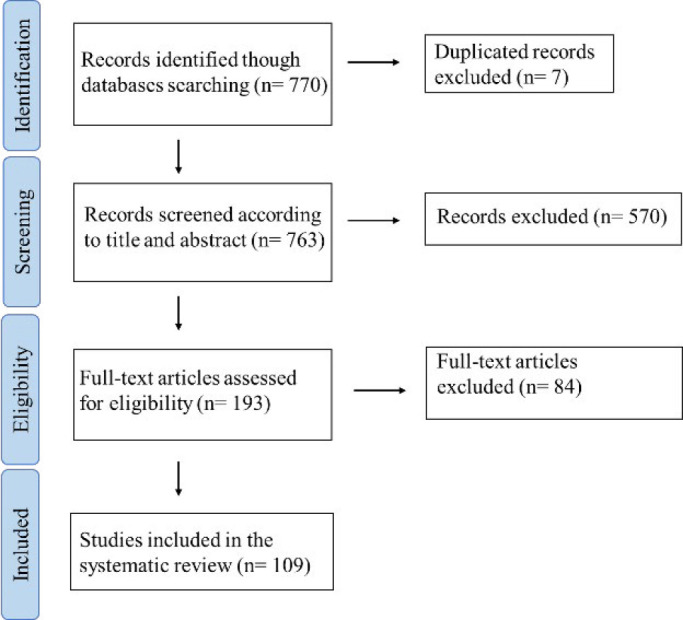
PRISMA flow diagram for screening of articles published between 2010 and July 2023

The process of screening records is shown in the PRISMA flowchart in Figure 2. The search identified 770 studies from PubMed and Scopus databases, of which 763 were screened for title and abstracts after removing duplicates. Inclusion and exclusion criteria are defined in Table 1. From the remaining 193 studies that were screened, 18 were excluded because their full text was not accessible. Finally, 84 articles were excluded, and 109 were assessed for eligibility.

### Study characteristic

The included studies are classified into the effect of different categories of phytochemicals on different cell lines. Characteristics of the included studies are summarized in the supplementary file (Table 2). It is worth mentioning that the 3T3-L1 mouse preadipocyte cell line is one of the most widely used models for studying the molecular mechanism of adipogenesis. Human visceral adipose tissue (vASCs), mouse C3H10T1/2, bone marrow-derived human mesenchymal stem cells (hMSCs), and human Monocytic Leukemia Cells (THP-1 cells) are the other cell lines in this article that have been less studied.

### Risk of bias studies

The QUIN tool criteria include the following items: clearly stated aims/objectives, detailed explanation of sample size calculation, detailed explanation of sampling technique, details of the comparison group, detailed explanation of methodology, operator details, randomization, method of measurement of outcome, outcome assessor details, blinding, statistical analysis, and presentation of results. All questions were answered separately. All studies assessed were unclear regarding the risk of bias. Most studies were rated as low risk for clearly stated aims/objectives, details of the comparison group, statistical analysis, and presentation of results. However, other criteria were unclear for most studies. Because of the large number of articles, the related Table is not attached.

## Anti-adipogenic effects

Many studies have been conducted on the anti-adipogenesis effects of phytochemicals and different plants. Examples include resveratrol, quercetin, apigenin, carvacrol, etc. In this article, we systematically investigated the effects of phytochemicals and herbs on inhibiting or reducing the process of adipogenesis leading to fat loss. The main cell lines studied were 3T3-L1 mouse preadipocytes and human adipose tissue, yielding acceptable. In most articles, cytotoxicity was first tested in the culture medium to determine the appropriate dose. Oil red o staining test was performed to quantify lipid accumulation, and real-time PCR was reliable for determining the transcription factors involved (20). Our study focused on investigating phytochemicals and plants that inhibit or reduce the process of adipogenesis. 

## Anti-adipogenic effects of phytochemicals and herbs in 3T3-L1 cells

The *in vitro* models are used to understand the adipogenesis process and explore new insights into the mechanism of therapeutics. One of the most important cell lines for studying adipogenesis is 3T3-L1 preadipocyte cells. Numerous researchers have assessed the effects of different phytochemicals and herbs on adipogenesis in 3T3-L1 preadipocytes. According to the San *et al*. study, pinostrobin at 5-20 µM for 48 hr significantly reduced intracellular lipid accumulation in mouse (3T3-L1) preadipocytes compared with the control group. At the same concentration, it was more effective than oxyresveratrol, a known anti-adipogenic compound. Pinostrobin, as a flavanone, is a major constituent of Thai fingerroot (*Boesenbergia rotunda*), accounting for about 1.2% w/w. Results showed that pinostrobin significantly decreased C/EBPα, PPARγ, SREBP-1c levels, and cellular triglyceride content in 3T3-L1 cells. In addition, pinostrobin at 10 and 20 μM reduced p-Akt/Akt and p-GSK3β/GSK3β levels in 3T3-L1 cells. In addition, it can excite the AMPK-acetyl-CoA carboxylase (ACC) signal via up-regulated p-AMPKα/AMPKα and p-ACC/ACC levels. Treatment with pinostrobin inhibited the expression of MAPK signaling molecules, including JNK and p38, in differentiated 3T3-L1 cells (21). Another study determined that administration of *Abeliophyllum distichum* Nakai leaf extract at a concentration of 50 and 200 μg/ml for 8 days significantly affected the expression of transcription factors involved in early adipogenic stages. *A. distichum* is a Korean endemic plant that is a source of several beneficial natural compounds with anti-oxidant, antidiabetic, anticancer, and antihypertensive properties (22). *A. distichum* extract significantly decreased lipid accumulation in differentiated 3T3-L1 adipocytes via reducing PPARγ, FAS, ACC, C/EBPα, SREBP1c, LPL, and AP2 expression levels. In addition, *A. distichum* extract also reduced the number of cells entering mitosis, suggesting a possible role in inhibiting cell proliferation (23). 

*Gymnema inodorum *has been used traditionally in central and southern India as an antidiabetic, hypoglycemic agent. The gymnemic acids (GiA-7), stephanoside C, and stephanoside B (100-µM; for 8 days) contained in *G. inodorum* tea could contribute to the prevention of obesity by inhibiting adipocyte differentiation and promoting beige adipocytes. Treatment with these compounds suppressed the expression of Lipin-1, PPARγ, C/EBPα, FASN, CD36, and fatty acid binding protein 4 (FABP4) genes in 3T3-L1 preadipocytes. Also, only GiA-7 increased the expression of UCP1 and PGC1α in 3T3-L1 preadipocytes (24). It was reported that anthocyanins (50, 100, and μg/ml for 8 days) can attenuate the terminal differentiation of 3T3-L1 preadipocytes by reducing the lipid content, number of lipid droplets, and triglyceride production. In addition, following treatment with anthocyanins, PPARγ, C/EBPα/β, and SREBP-1c expression level and gene expression of AP2, leptin, and FAS were inhibited. Additionally, it was shown that anthocyanins could activate the AMPK signaling by enhancing the phosphorylation of AMPK and ACC during 3T3-L1 differentiation. Anthocyanins are one of the most important water-soluble polyphenolic pigments extracted from the fruit of *Vitis coignetiae*. *V. coignetiae *Pulliat belongs to the genus Vitis and has been used in traditional medicine to treat various diseases, including inflammatory disorders, liver disorders, cancer, and cardiovascular diseases (25). The evaluation of the euphorbiasteroid (12.5, 25, and 50 μM for 8 days) on adipogenesis of 3T3-L1 cells effects demonstrated that euphorbiasteroid anti-adipogenic effects could be attributed to the inhibition of signals involved in the initial phase of adipogenesis and the activation of anti-adipogenic proteins and gene expressions. Results showed that euphorbiasteroid increased the phosphorylation of AMPK and ACC, which altered the key regulator proteins of adipogenesis in the early stage. In contrast, treatment with euphorbiasteroid decreased FAS, PPARγ, and C/EBPα protein levels at the late stage of adipocyte differentiation (26). 

Antofine, a phenanthroindolizidine alkaloid derived from the root of *Cynanchum paniculatum* Kitagawa, is extensively used for pharmacological effects, including anti-inflammatory, antitumorigenic, and antiviral activities. The results of a study by Jang *et al*. using an *in vitro* model also support the anti-adipogenic effects of antofine. They found that, by inhibiting PPARγ expression and AP2 promoter activity, antofine (10 nm, 48 and 96 hr) effectively attenuated PPAR-induced adipogenic gene expression. This inhibited adipocyte differentiation, implying that antofine can prevent the maturation of preadipocytes into mature adipocytes (27). Furthermore, the effect of *Cyclopia* on adipogenesis in 3T3-L1 preadipocytes has been the subject of the study by Dudhia *et al*. They concluded that hot water extracts of *C. maculata* and *C. subternata* (20-1600 µg/ml, 8 days) significantly suppressed triglyceride content and lipid accumulation in 3T3-L1 cells. Also, the plant extracts, especially fermented *C. maculata* and *C. subternata*, showed promising effects in altering the expression of PPAR isoforms in 3T3-L1 adipocytes (28). Similarly, another study reported that water extract and acetic ether extract of *Sibiraea angustata* (50, 100, and 200 mg/ml, 7 days) effectively inhibited adipocyte differentiation by negatively regulating key adipogenic transcription factors C/EBPβ PPAR, as well as AP2, LPL and GLUT-4, inhibiting PPAR transcriptional activity, and affecting cell cycle proteins involved in mitotic clonal expansion (MCE) (29). 

The anti-adipogenesis effect induced by 3-O-β-D-glucopyranoside has been demonstrated in two studies. Quercetin-3-O-(600-feruloyl)-b-D-galactopyranoside (10 µm, 9 days) demonstrated a temporal suppressive effect on adipogenesis in 3T3-L1 cells. The compound specifically inhibits adipocyte differentiation via inhibiting mRNA and protein expression of PPAR, C/EBPα when administered during the critical period from day 2 to day 6 of induction (30). In addition, in another study, isorhamnetin 3-O-β-D-glucopyranoside (20 µm, 6 days) impeded lipid accumulation, down-regulated key transcription factors PPARγ, C/EBPα, and differentiation- SREBP1 and adipogen-specific proteins FAS, GLUT 4, retinoid X receptor (RXR)α, and leptin, and activated AMPK (31). Another investigation assessed the anti-adipogenic efficacy of (+)-Episesamin extracted from *Lindera obtusiloba* using 3T3-L1 (pre)adipocytes. Results showed that (+)-epi sesamin (10 µm, 8 days) revealed direct anti-adipogenic effects on preadipocytes and mature adipocytes, possibly through modulation of Wnt, PPAR, iNOS, and ERK1/2 signaling pathways. Moreover, it showed an anti-inflammatory effect by decreasing the TNFα and IL-6 (32). In accordance with the findings of other studies, Gaya and colleagues indicated that the carnosic acid isolated from Rosmarinus officinalis *as a main bioactive compound* considerably inhibits adipocyte differentiation in 3T3-L1 preadipocytes. The exact mechanisms of action of carnosic acid (10, 20, 30 µg/ml, 6 days) are the down-regulation of the expression of PPARγ and FABP4 and alteration of the subnuclear distribution of C/EBPβ. Increasing the LIP/LAP ratio has been identified as a possible mechanism contributing to the anti-adipogenic effects of these compounds (18). In a study, Kang and colleagues examined and compared the *Sasa quelpaertensis* Nakai extract (125, 250, 500 µg/ml, 8 days) and *p*-coumaric acid (its main compound) (12.5, 25, 50, 100 µM, 8 days) impacts on adipogenesis in 3T3-L1 cells. *S. quelpaertensis* Nakai extract inhibited adipogenesis by down-regulating key adipogenic transcription factors C/EBPα, PPARγ, SREBP-1c, and aP2, also decreasing FAS and adiponectin mRNAs expression through the activation of AMPK in the early phase of differentiation. On the other hand, *p*-coumaric acid exerted its anti-adipogenic effects in the late phase of differentiation by attenuating the expression of adipogenic transcription factors. Additionally, *p*-coumaric acid promoted beta-oxidation of fatty acids in mature adipocytes via activation of AMPK (33). *Acanthopanax henryi* (Oliv.), a traditional Chinese herb, has been used in various pathologic conditions such as arthritis, paralysis, lameness, rheumatism, edema, hernia, injury due to falls, and abdominal pain. Han and colleagues investigated the effects of Lycosides St-C1 and glycosides St-E2 isolated from the leaves of *A. henry *(Oliv.) Harms on adipocyte differentiation in 3T3-L1 cells. The expression PPAR and C/EBPα was suppressed, and phosphorylation of AMPK increased in 3T3-L1 cells following treatment with Glycoside St-C1 and Glycoside St-E2 (0.5 and 1 μg/ml for 6 days) (34). 

Another investigation determined that fucosterol isolated from *Ecklonia stolonifera* at 25 and 50 μM concentrations for 8 days did not exhibit significant cytotoxicity at lower concentrations and showed anti-adipogenic effects on 3T3-L1 preadipocytes. Its effects were related to the down-regulation of key adipogenic genes (PPAR, C/EBPα, and SREBP-1) and suppression of PI3K/Akt and ERK signaling pathways. In addition, fucosterol inhibited the phosphorylation of FoxO1, resulting in the activation of FoxO1 and suppression of adipocyte differentiation (35). It was interesting that *Monascus ruber*-fermented *Fagopyrum esculentum* (red yeast buckwheat, RYB) at 50–800 μg/ml for 6 days can inhibit 3T3-L1 cell differentiation and PPARγ, C/EBPα expression, and aP2, FAS, and leptin gene expression during adipocyte differentiation. RYB treatment affected cell cycle regulators. The expression of CDK2 and cyclin D decreased in a dose-dependent manner, whereas the expression of p21 and p27 increased in response to RYB treatment. These changes suggest that RYB may affect cell cycle progression and inhibit cell proliferation (36). 

Zyflamend, a well-defined, unique blend of ten natural herbal extracts, revealed beneficial effects such as anti-inflammatory and antitumorigenic. Interestingly, Zyflamend showed a great anti-adipogenic impact on white 3T3-MBX preadipocytes. Treatment with 200 μg/ml for 12 days with Zyflamend attenuated proliferation and inhibited lipid accumulation and expression of lipogenic genes; it also increased cell lipolysis and death of 3T3-MBX preadipocytes. Results showed that Zyflamend significantly decreased FASN, PCB, C/EBP, adiponectin, and PPARγ expression in 3T3-MBX cells. Also, it can reduce the expression of Glut4 and insulin-stimulated glucose uptake. Mechanistically, Zyflamend can affect adipogenesis through modulation of AMPK, PKA, and JNK. Inhibition of JNK or PKA fully restored adipocyte differentiation, whereas inhibition of AMPK only partially restored adipogenesis, suggesting the involvement of pathways in the effects of Zyflamend (37). Similarly, Hadrich *et al*. reported that apigetrin treatment (100 μM, for 8 days) exerted anti-adipogenic effects via suppressing C/EBP-α, PPAR-γ, SREBP-1c, and FAS mRNA levels in 3T3-L1 preadipocytes during the early stages of differentiation. Also, it was shown that apigetrin treatment inhibited the mRNA level of pro-inflammatory genes (TNF-α and IL-6) and effectively attenuated H_2_O_2_-induced production of ROS in adipocytes. Apigetrin, known as apigenin 7-O-glucoside, is a glycosyloxyflavone found in many plant leaves and seeds with medicinal benefits such as anti-oxidant, anticancer, and anti-inflammatory properties (38). In addition, in a study, Spalletta *et al*. evaluated the anti-adipogenic effect of carvacrol in the murine 3T3-L1 cell lines. Carvacrol is a phenolic monoterpenoid found in various plant essential oils with different effects, including antimicrobial, anti-inflammatory, anti-oxidant, analgesic, anti-carcinogenic, anti-proliferative, and antiplatelet. Results showed that carvacrol at 25 μM reduced cell differentiation in 3T3-L1 (40%; after 7 days) cell lines by analysis of LC3-II levels (16). Kaempferol is a flavonoid found in various plants, especially *Solidago virgaurea* Nakai. *S. virgaurea* has been used as a food, stomachic acid, and diuretic in Europe and other parts of the world. Kaempferol-3-O-rutinoside treatment (10 μg/ml; for 8 days), extracted from whole grass of *S. virgaurea,* suppressed adipogenesis and differentiation of 3T3-L1 cells, most likely by down-regulating the expression of PPAR-γ and C/EBPα (39). In a study, Zhang *et al*. assessed the anti-adipogenic properties of berberine 3T3-L1 preadipocytes to find their molecular mechanisms. Berberine is a widely used medicinal plant constituent with several pharmacological properties for treating diarrhea, oriental sore, diabetes mellitus type 2, hypercholesterolemia, trachoma, and congestive cardiac failure. They found that berberine (5 μM; for 7 days) exerted its anti-adipogenic effect by inhibiting the mRNA expressions of C/EBPα, PPARγ2, SREBP1c, and LPL. Also, berberine suppressed CREB activity and subsequently decreased the expression of C/EBPβ, an early regulator of preadipocyte differentiation in 3T3-L1 cells (40). Interestingly, a study examined the effects of esculetin on apoptosis and adipogenesis in 3T3-L1 cells. Based on the results, esculetin at 50, 100, 200, 400, and 800 μM from 6 to 48 hr induced apoptosis in both preadipocytes and mature adipocytes. However, the timing and dosage of the effect differ between the two cell types. Preadipocytes required higher concentrations and a longer exposure time (48 hr) for apoptosis to occur, whereas mature adipocytes were more sensitive and showed increased apoptosis as early as six hours after treatment, even at lower esculetin concentrations. Also, at 100 and 200 μM concentrations, esculetin reduced viability during all periods of adipocyte differentiation (early, intermediate, and late stages). Esculetin (12.5, 25, 50, 100, or 200 μM; for 6 days) can also decrease lipid content in a dose-dependent manner 3T3-L1 preadipocytes (14). The evaluation of the coumestrol effects on adipogenesis demonstrated that coumestrol exerted its influence on early differentiation by decreasing MDI (mixture of 3-isobutyl-1-methylxanthine, dexamethasone, and insulin)-induced lipid accumulation. Treatment with coumestrol (20, 40 µM, 8 days) also suppressed PPARγ and C/EBPα protein expression in adipocytes, which led to the subsequent down-regulation of FAS and aP2 expression. In addition to the features mentioned, coumestrol exhibited its influence on early differentiation by modulating the Akt signaling pathway, leading to down-regulation of phosphorylated Akt and GSK3β. In addition, coumestrol affected the Wnt/β-catenin signaling pathway by reversing MDI-induced down-regulation of β-catenin, Wnt10b, and LRP6 (41). Apigenin showed two significant effects on 3T3-L1 adipocytes: it suppressed lipolysis by down-regulating the expression of hormone-sensitive lipase (HSL) and monoacyl glyceride lipase (MGL) genes. In contrast, the adipose triglyceride lipase (ATGL) gene was unaffected. Moreover, apigenin activated AMPK, an important regulator of adipogenesis, suggesting that this activation may contribute to the suppressive effect of apigenin on adipocyte differentiation (42). Dohongsamul-tang, a traditional herbal formula, is often used in ischemic heart and brain diseases (43). Interestingly, Dohongsamul-tang (62.5, 125, 250, and 500 μg/ml; for 7 days) effectively inhibited adipogenesis in 3T3-L1 cells by down-regulating adipocyte-specific markers such as PPARγ, C/EBPα and FABP4, and reducing lipid accumulation. It also leads to decreased production of adipokines such as adiponectin, resistin, and PAI-1 (44). Trigonelline, a pyridine alkaloid isolated from edible plants, especially *Trigonella Foenum-Graecum*, has numerous medicinal properties, including antimigraine, memory-improving, sedative, antiviral, and antibacterial. It is worth mentioning that trigonelline at 75 and 100 μM (on the 5th and 10th days of differentiations) showed anti-adipogenesis via reducing the PPAR and C/EBPα mRNA expression, which results in further reduction of other genes such as adipogenin, adiponectin, resistin, leptin, and AP2. The inhibitory effect of trigonelline on the mRNA transcripts of fatty acid synthase and GLUT-4 was comparable to the inhibitory effect of isoproterenol, a known inhibitor of fatty acid synthesis and glucose uptake (45). Similar to other phytochemical, soyasaponins Aa and Ab (25, 50, and 100 μM, 8 days) successfully suppressed the gene expression of two important transcription factors, PPAR and CEBPα, resulting in a significant reduction in the expression of several adipogenic marker genes, including adiponectin, ADD1/SREBP1c, AP2, FAS, and resistin in 3T3-L1 adipocytes (46). 

Parthenolide, a sesquiterpene lactone gathered from the feverfew plant (*Tanacetum parthenium*), has been used for medicinal purposes for a long time. A high concentration (8 μM) of parthenolide reduced the protein abundance of PPARγ and C/EBPα and its target protein FABP4 by 98, 96, and 99% during adipogenesis. Administration of parthenolide in the early stages of adipogenesis (0–2, 0–4, and 0–6 days) showed a 24%, 36%, and 50% reduction in lipid accumulation due to a reduction of the protein levels of C/EBPβ, a pro early adipogenic factor, and an increase in KLF2, an anti-early adipogenic factor (47). The effects of 7, 8-dihydroxyflavone, a naturally occurring flavone found in plants such as primula tree, in 3T3-L1 cells were investigated by Choi *et al*. Treatment with 7, 8-dihydroxyflavone (1, 10, and 20 μM) every 2 days for 8 days significantly suppressed lipid accumulation as a result of massive reduction in the expression of PPARγ, aP2, C/EBPβ and its mediated protein, C/EBPα. Additionally, 7, 8-dihydroxyflavone down-regulates the MAPK signaling pathway by inhibiting the phosphorylation of p38 and ERK, which are MAPK proteins necessary to accelerate the differentiation of preadipocyte cells (48).

*Orostachys japonicus* reduced the production of reactive oxygen species (ROS) during adipogenesis and significantly decreased the mRNA and protein expression of NOX4 and G6PDH, and increased SOD-1 and SOD-2 protein levels, confirming its ability to inhibit the formation of ROS and resist oxidative stress (49). Mulberry (*Morus alba* L.) has long been used as a medicinal plant, especially in Asian countries. Mulberry is loaded with carbohydrates, vitamins, proteins, and fiber and has a low-calorie content (50). Treatment with mulberry fruit extract at 50 and 100 μM for 7 days significantly decreased lipid and triglyceride accumulation and GPDH activity and reduced the expression levels of PPARγ, C/EBBPα, and AP2 along with miR-21 and miR-143 in 3T3-L1 cells (51). In the presence of DMII (differentiation medium), increased Akt phosphorylation at Thr-473 indicated the activation of the Akt pathway. However, treatment with centipede grass (10, 100 µg/ml, 6 days) decreased Akt phosphorylation, suggesting that centipede grass inhibits Akt activation during adipocyte differentiation. Also, centipede treatment inhibited C/EBPβ, C/EBPα, and PPARγ expression in 3T3-L1 preadipocytes (52). Buckwheat sprout (TBWE) treatment (50 μg/ml, 8 days) significantly decreased the production of ROS in fully differentiated 3T3-L1 cells during adipogenesis. This suggests that TBWE has anti-oxidant effects on adipocyte differentiation. TBWE significantly decreased the expression of pro-oxidant enzymes such as NOX4 and the NADPH-producing enzyme G6PDH. Furthermore, the levels of the anti-oxidant enzymes GPx and Cu/Zn SOD were also lower in the TBWE-treated group compared with the control group, suggesting that TBWE may regulate lipid accumulation and the production of ROS by affecting these enzymatic pathways. Also (TBWE) treatment decreased C/EBPα, PPAR γ, and aP2 levels in 3T3-L1 preadipocytes (53). Mogrol (20 μM) suppressed cellular triglyceride levels when administered during the early (days 0–2) and late (days 4–8) phases of adipocyte differentiation but not during the middle phase (days 2–4). The suppressive effect was stronger in the early phase than in the late phase and decreased cellular DNA levels, indicating a reduction in cell division (clonal expansion) when exposed to stimuli during the early differentiation phase (54). 

Baicalein is an abundant plant flavonoid present in vegetables and fruits. Protein expression of key adipocyte-related factors, including PPARγ, C/EBPα, FAS, SCD, and GLUT-4, was decreased by baicalein. Treatment with baicalein (50 μM, 6 days) significantly decreased glycerol release from adipocytes and the Akt signaling pathway (55). *Ecklonia cava* is an edible marine brown algal, rich in polyphenol components mostly settled in Korea and Japan (56). Treatment with enzyme-treated *E. cava* extract (50 μg/ml, 24 hr) resulted in a dose-dependent reduction in glucose utilization and TG accumulation. It also decreased the expression of C/EBPα significantly, but not C/EBPβ and PPARγ (57). Guarana (100, 150, 200, and 300 µg/ml, 96 hr) suppressed the expression of specific miRNAs, including mmu-miR-27b-3p, mmu-miR-34b-5p, and mmu-miR-760-5p. The down-regulation of these miRNAs contributed to the up-regulation of their target genes Wnt3a, Wnt1, and Wnt10b, which are known to inhibit adipogenesis (58). TBE (tartary buckwheat extract) (100 µg/ml, Up to 7 days) significantly reduced GPDH activity in 3T3-L1 adipocytes and decreased mRNA levels of inflammatory mediators, including TNF-α, IL -6, MCP-1, and iNOS. The anti-inflammatory effect of TBE may be due to the presence of compounds such as rutin and other phenolic acids found in tartar buckwheat. Rutin is associated with anti-inflammatory properties and has been shown to be useful in various inflammatory conditions (35). BG (Black Ginseng) and ginsenoside Rb1 have differential effects on adipogenic markers during differentiation of 3T3-L1 cells and PWATs (primary white adipocytes). BG increased PPAR expression and inhibited C/EBPα and SREBP-1c in both adipocyte types in a dose-dependent manner, whereas Rb1 up-regulated PPAR but showed dose-dependent inhibition of C/EBPα and SREBP-1c expression (59). Saikosaponin A (SSA) and Saikosaponin D (SSD) from the root of Brachyponera chinensis down-regulated adipogenesis and inhibited lipid accumulation in 3T3-L1 cells. They achieve this by suppressing the expression of key adipogenic transcription factors (PPAR, C/EBPα, and Srebf1), associated lipogenic genes (FABP, FASn, and LPL), and by activating the AMPK pathway. Besides, the MAPK pathway is likely involved in the anti-adipogenic effects of SSA and SSD (60). 6-Gingerol was shown to inhibit adipogenic differentiation in 3T3- L1 cells by reducing lipid accumulation and down-regulating the adipogenic markers PPAR-γ and C/EBP-α. Its mechanism was activating the canonical Wnt/β-catenin pathway, which promotes nuclear translocation of β-catenin and leads to inhibition of gene expressions associated with adipogenesis (61). Treatment with ethyl acetate extract of the flower of *Edgeworthia gardneri* notably increased the levels of phosphorylated AMPK and phosphorylated ACC in a concentration-dependent manner (62). *Andrographis paniculata* is a popular herbal medicine planted in China, Taiwan, and Southeast Asia. It is used to treat diarrhea, sore throat, colds, and liver diseases. Andrographolide is the main diterpenoid of *A. paniculata*. Andrographolide treatment (7.5, 15 µM, 8 days) resulted in a dose-dependent increase in the proportion of cells arrested in the G0/G1 phase, indicating cell cycle arrest at the mitotic clonal expansion stage. It also inhibited PKA activation and subsequently decreased CREB phosphorylation, resulting in down-regulation of C/EBPβ expression (63). CBMG (chlorobenzoyl mansonone G) has been isolated from *Mansonia gagei*, a traditional medicine as a cardiac stimulant. Treatment with 10 μM of CBMG regulated protein expression of both PPAR and C/EBPα, but interestingly, only C/EBPα gene transcription was affected, whereas PPAR mRNA levels remained unchanged. This suggests that CBMG may affect translational or posttranslational modifications of PPAR to decrease its protein expression. In addition, CBMG inhibited the DNA-binding activity of PPAR, resulting in a decrease in PPAR-mediated gene promoter activity. However, the exact mechanisms of the effects of CBMG on PPAR activity and DNA binding remain to be elucidated (64). Rutin (Quercetin-3-O-rutinoside) is found in many fruits, vegetables, and beverages. In a study, Ganjayi *et al*. isolated rutin, a flavonol glycoside, from *Moringa oliefera* and investigated its anti-adipogenesis effects. Interestingly, treatment with 10, 20, and 40 μM of rutin showed inhibitory activities on digestive enzymes, α glucosidase, and pancreatic lipase. The mRNA expression levels of UCP- 1 and GLUT- 4 were enhanced, and PPARγ was reduced, but AMPK remained unchanged in rutin 3T3- L1 treated cells. Whereas a down-regulation of PPARγ and an up-regulation of UCP-1, p- AMPK, and GLUT-4 protein expression were observed. This result showed that rutin markedly increased glucose uptake and inhibited adipogenesis (65). Lim and colleagues investigated the effects of EH-CS (*Ephedrae herba* and *Coicis semen* herbal pair) on adipogenesis in 3T3-L1 cells. AMPK phosphorylation/AMPK was significantly increased in EH-CS compared with differentiated adipocytes. In the early differentiation phase (24 hr), adipocytes showed a significant increase in the expression of PPAR, FABP4, and CEBP genes; treatment with EH-CS samples significantly reduced this increase. The expressions of SCD1, FASN, ACC1, and SREBF1 were suppressed by treatment with EH-CS (66). *Withania somnifera, *generally recognized as Indian ginseng, has been used as a functional food to promote health and longevity and maintain and restore health. Lee *et al*. conducted a phytochemical exploration of the MeOH extract of Indian ginseng. Results showed that a concentration of 25 μM compounds inhibited adipogenesis and suppressed the enlargement of lipid droplets compared to control. Furthermore, the mRNA expression levels of Fabp4 and Adipsin were markedly reduced. They also observed that Indianseng compounds regulated lipid metabolism by up-regulating the expression of the lipolytic genes, ATGL and HSL, and down-regulating SREBP1, the lipogenic gene (67). *Cirsium brevicaule *A GRAY (CBAG) is a wild perennial herb native to Southern Japan and China. Leaves, stems, and roots of CBAG are used as both food and herbal medicine on Japanese islands. A chemical structure named syringin was identified from the CBAG root by Hossin *et al*. They evaluated the lipid metabolism related genes to understand the molecular mechanisms related to syringing. Treatment with syringin at 5, 10, and 20 μM decreased the mRNA levels of lipogenic-related genes SCD1 and GLUT4. On the other hand, syringin significantly increased the expression levels of LIPE and CPT1a, lipolysis-related genes. Further, syringing lowered PPARγ, FABP4, and ADIPOQ compared with untreated 3T3-L1 cells. Syringin also increased the levels of AMPK and resulted in the phosphorylation of ACC, which in turn inhibits adipogenesis (68).

According to the study by Sung *et al*., BS21 is the most effective combination of *Phyllostachys pubscenens* leaves and *Scuttellaria baicalensis* roots. They showed that treatment with 60, 120, 240, and 480 μg/ml BS21 significantly reduced the differentiation and accumulation of adipocytes and the size of lipid droplets. The secretion of adipokines, leptin, and adiponectin was also reduced by 480 μg/ml BS21. At the molecular level, 7-day treatment with BS21 decreased protein expression of PPARγ, C/EBPα, ap2, and the lipogenic markers SREBP1c and FAS. BS21 at doses of 240 and 480 μg/ml increased AMPK phosphorylation, resulting in regulation of the expression of the brown fat proteins UCP1, PRDM16, and PGC1, suggesting that BS21 regulates lipid metabolism in 3T3-L1 adipocytes (69). *Chrysanthemum morifolium* is a perennial plant known in China for medicinal and ornamental purposes. Important ingredients such as apigenin, lutein, and chlorogenic acid have been found in *C. morifolium*, which have anti-obesity effects. Lee and Kim investigated the anti-adipogenic effect of the hot water extract of *C. morifolium* in 3T3-L1 adipocytes. The results showed that lipid and TG levels were inhibited in the presence of 1 μg/ml HCF compared to control cells. GPDH activity was significantly reduced in HCF-treated cells. In addition, HCF suppressed the mRNA levels of PPARγ, CEBPα, SREBP-1c, FABP4, ACC1, and FAS in a dose-dependent manner after 7 days. Further investigations showed that AMPK and SIRT1 activity was increased after HCF treatment (70). In rats fed high-fat diet, Taurine reduced the fat deposition via chnge in FAS and ATGL content (6). *Momordica cochinchinensis* is native to South Asia. The plant is known in various regions as gac, spiny bitter gourd, laurel fruit, and red melon. Yu *et al*. carried out a phytochemical study of the seeds of *M. cochinchinensis* and isolated two triterpenoid saponins: gypsogenin 3-O-β-d-galactopyranosyl (1→2)-[α-l-rhamnopyranosyl(1→3)]-β-d glucuronopyranoside and quillaic acid 3-O-β-dgalactopyranosyl(1→2)-[α-l rhamnopyranosyl(1→3)]-β-d-glucuronopyranoside with the most significant effect on adipocytes. After testing the viability of the cells, compound 1 was selected for further experiments. The result showed that compound 1 strongly inhibited lipid accumulation at a concentration of 100 μM. The gene and protein levels of adipogenic markers were measured on day 4 of differentiation. They showed that the gene expression of C/EBPα and PPARγ and the protein expression of C/EBPα, PPARγ, and FABP4 decreased significantly after treatment with compound 1. Treatment with compound 1 dramatically suppressed the gene expression of HSL and ATGL, which led to the inhibition of lipolysis compared to the control group (71). Eriocalyxin B is an en-kaurene diterpenoid from a Chinese herb, *Isodon eriocalyx*. The compound has been shown to possess multiple anti-inflammatory and anticancer properties. Mu *et al*. investigated the effects of eriocalyxin B on adipogenesis. Treatment with 2.5 μM of eriocalyxin B blunted the adipogenesis process at the early stages significantly. Furthermore, the result displayed that the cells aggregated in the G2/M phase during the MCE period of adipogenesis after treatment with the compound compared to the control group. To prove this fact, the levels of the key cell cycle regulators in the G2/M phase were measured. They found that the CDK1, Cyclin A, and B mRNA levels were suppressed by eriocalyxin B at 24 hr after differentiation (72). 

*Allium sativum* L. (garlic) is one of the spreading plants in the Amaryllidaceae family. Garlic is used worldwide as a functional food and is known for its effects against cancer, fever, headaches, intestinal worms, etc. Baek *et al*. isolated six compounds, three eugenol diglycosides and three β-caroline alkaloids, from methanolic (MeOH) garlic extract and investigated the effects on adipocyte metabolism. 3T3-L1 cells were treated with 20 μM of all compounds during adipogenesis. Staining lipid droplets with Oil Red O solution showed that these compounds inhibited lipid accumulation. The transcript levels of FABP4, PPARγ, C/EBPβ, Adipsin, and Adipoq were also reduced by treatment with all compounds. Compound 6 showed the strongest effect among compounds 1-6, both in the early stages and throughout the process of adipogenesis. Treatment with compound 6 significantly inhibited lipid accumulation, even at a low concentration (5 μM). In addition, mRNA expression of the lipogenic gene SREBP1 was significantly reduced under the action of compound 6. In contrast, transcription of the lipolytic genes ATGL and HSL was increased, representing a potential therapeutic agent to prevent excessive adipogenesis in obesity (73). Oh *et al*. tested solvent-based fractions of *Artemisia princeps* for their ability to inhibit adipogenesis. *A. princeps*, the Korean mugwort, is an edible plant with various health benefits and an important ingredient in traditional medicinal recipes. This study showed that all tested fractions could inhibit lipid accumulation at a 50 μg/ml dose. Also, all of the fractions could suppress the expression of mRNA and protein levels of PPARγ, C/EBPα, and SREBP-1. On the other hand, results showed a decrease in MAPK pathway protein activation, especially p-38 and ERK, which decreased the expression of PPARγ and C/EBPα (74). The butterfly pea, or *Clitoria ternatea* L., is a common plant in South and Central America. This herb has long been used to cure various conditions, including fever, constipation, stomach pain, snakebite, scorpion stings, and eye ailments. A study on *C. ternatea* flower extract (CTE), which mostly comprises anthocyanins generated from delphinidin, was carried out by Chayaratanasin and his team. At 250, 500, and 750 μg/ml concentrations, CTE considerably reduced the G2/M phase and increased the cell distribution in the G0/G1 phase, delaying the cell cycle. The findings showed that, compared to the control, CTE significantly decreased the phosphorylation level of Akt1 and repressed the phosphorylation of ERK1/2. Treatment with CTE (500–1000 μg/ml) markedly reduced the formation of triglyceride and lipid droplets. PPARγ and C/EBPα mRNA and protein levels were also decreased by CTE, in that order (75). *Betula platyphylla* var. *japonica*, also known as Asian white birch, is distributed throughout Asian countries, including Japan, Korea, and China. The bark of this plant has been used for the treatment of a wide variety of inflammatory diseases, such as choloplania, pneumonia, chronic bronchitis, and dermatitis, as well as for the relief of heat and cough. In the Huh *et al*. study, 3T3-L1 preadipocytes were treated with phenolic compounds isolated from the EtOH extract of the bark of *B. platyphylla* var*. japonica*. The compound was treated with 3T3-L1 cells at 50 and 100 μM doses at the start of differentiation until the fourth day. The result showed that phenolic compounds down-regulated FABP4, PPARγ, and C/EBPα levels in a dose-dependent manner, where at 100 μM, the markers were fully suppressed, which would be beneficial in losing weight (76). Sharma and his group tested *Garcinia cambogia* extract (GE) and *Pear pomace* extract (PE) to see if the mixture of PE and GE (MIX) had a more effective anti-adipogenic activity than PE or GE alone. Hydroxycitric acid (HCA), an active ingredient from *G. cambojia*, inhibits adenosine triphosphate citrate lyase required for fatty acid biosynthesis. The pear contains phenolic compounds that prevent hyperglycemia and dyslipidemia. Cells treated with 30 μg/ml PE slightly inhibited lipid accumulation. Treatment with 60 μg/ml GE showed significant inhibition of adipocyte differentiation. However, cells treated with MIX showed a stronger inhibition of lipid accumulation. Treatment with PE, GE, and MIX during the differentiation phase suppressed the expression of C/EBPα, with MIX showing the greatest inhibitory effect. Similarly, treatment with GE and MIX significantly reduced the expression of PPARγ. The expression of FAS was decreased after the addition of PE GE or MIX. Also, adding PE, GE, and MIX enhanced the HSL expression. The combination of PE and GE enhanced HSL expression even further, suggesting greater anti-adipogenic and lipolytic activity (77). *Aster yomena*, a traditional Korean plant, is used to treat diseases such as asthma, coughs and insect bites. In a study, Han *et al*. investigated the effect of the ethanol extract of *A. yomena* (EEAY) against obesity on 3T3-L1 preadipocytes. EEAY reduced cell size and the number of lipid droplets in mature adipocytes in a concentration-dependent manner (50-200 μg/ml). Furthermore, the results showed that inhibiting lipid accumulation by EEAY was associated with suppressing PPARγ, C/EBPα/β, and SREBP-1c expression. After EEAY administration, the expression of aP2 and leptin, which are involved in lipid metabolism, was decreased in a concentration-dependent manner. EEAY significantly increased the phosphorylation of AMPK and ACC, suggesting that the inhibitory effect of EEAY on adipogenesis in 3T3-L1 cells may be achieved by activating the AMPK signaling pathway (13). *Hibiscus rosa sinensis* is an ornamental plant cultivated in India and China and has many medicinal properties such as wound healing, anti-infertility, antihypertensive, hypolipidemic, anticancer, and cardio-protective activity. Lingesh *et al*. investigated the effect of ethyl acetate extract of *H. rosa sinensis* flower (HFR) at 25 and 50 μg/ml concentrations in 3T3-L1 cells. Lipid accumulation and cellular triglyceride content were decreased at these concentrations, respectively. Quantitative real-time PCR showed that HFR treatment significantly down-regulated gene and protein expression of PPARγ, C/EBPα, SREBP-1c, and FABP4. However, adiponectin expression was up-regulated. HFR dose-dependently increased the protein concentration of pAMPK, suggesting that the anti-adipogenic activity of HFR is mediated by activation of the AMPK signaling pathway (78). Do In Seung Gi-Tang (DISGT) is a herbal medicine containing the extracts of five different herbs traditionally used to treat blood stasis syndrome. Shim *et al*. tested 62.5, 125, 250, and 500 μg/ml concentrations of this formula on the process of adipogenesis. Treatment with various concentrations of DISGT significantly suppressed lipid accumulation and TG release in a dose-dependent manner. DISGT also suppressed the release of adipokines, adiponectin, and resistin. As described above, the expression of PPARγ and C/EBPα was decreased compared to the control group (79). *Camellia ptilophylla *(cocoa tea) is a naturally decaffeinated tea plant belonging to the *Camellia* genus. It has been widely consumed by local inhabitants in China. It contains theobromine and (-)-gallocatechin gallate (GCG). Li and his group investigated the influence of cocoa tea extract (CTE) and green tea extract (GTE) on adipogenesis. Microscopic observation showed that at 50 μg/ml, CTE and GTE significantly reduced oil droplet formation and lipid accumulation. Both CTE and GTE treatments decreased the expression of PPARγ and C/EBPα. The increase in the mRNA levels of adipocyte-specific genes SREBP-1c, SCD-1, ACC, FAT, and FAS after maturation was reversed by CTE and GTE. The MAPK pathways play an important role in signaling the expression of C/EBPα and PPARγ. CTE treatment markedly decreased the phosphorylation of ERK, p38, and JNK. Taken together, the result showed that CTE and GTE inhibited the adipogenic differentiation of 3T3-L1 cells (80). Kim *et al*. examined the effects of the aqueous methanol extract of *Porphyra yeoensis* (laver) on adipogenesis and apoptosis in 3T3-L1 cells. Laver is one of the most widely consumed red algae. Treatment with laver extract at 5, 10, and 15 mg/ml decreased the expression of all adipogenic proteins, particularly C/EBPα, FABP4, and FAS. Results indicated that treatment with laver extract did not cause significant changes in apoptosis of preadipocytes. In contrast, the differentiated adipocytes exhibited increased apoptosis when treated with laver extract at concentrations of 10 mg/ml and above. Laver extract treatment also reduced the GSH/GSSG ratio and total glutathione content (81). Jeong *et al*. conducted an *in vitro* study on a traditional Korean herbal formula called samsoeum (SSE). SSE comprises 12 medicinal herbs and is used to treat the common cold, fever, and headaches. Jeong *et al*. found that SSE-treated cells had noticeably less intracellular lipid droplet and TG accumulation at 50–400 μg/ml concentrations. Furthermore, leptin production was inhibited by SSE. SSE treatment reduced the mRNA expression of PPARγ and C/EBPα. In particular, SSE caused greater PPARγ expression suppression and decreased its target genes FAS, LPL, and FABP4 in 3T3-L1 adipocytes. Jeong *et al*. conducted a western blot analysis to examine whether SSE treatment could influence the MAPK pathways. SSE increased the level of phospho-ERK1/2. In contrast, SSE had no significant effect on p38 MAPK or JNK in the cells, suggesting the anti-adipogenic effects of SSE besides its other beneficial properties (82). *Coccinia grandis* L. Viogt (ivy gourd) is a tropical plant widespread in Africa, Asia, and the Pacific islands. Southeast Asians have long used this plant in their local cuisine and traditional medicine. Bunkrongcheap *et al*. extracted dried roots, stems, and leaves of ivy gourd with ethanol and applied it to 3T3-L1 preadipocytes. The results showed that the root extract at a 100 μg/ml concentration suppressed intracellular lipid accumulation only in the early phase of adipogenesis. In contrast, extracts from leaves and stems did not affect adipocyte differentiation. When they examined the effects of root extract on the expression of key activators of adipogenesis, both mRNA and protein levels of PPARγ and C/EBPα were significantly reduced. As a result of PPARγ inhibition, the expression of adiponectin and GLUT4 was reduced, suggesting that ivy gourd root is most effective in inhibiting adipogenesis (83). *Rhus verniciflua* stokes (RVS) is used as a traditional herbal medicine with anticancer, anti-oxidant, anti-inflammatory, antiplatelet, antifibrotic, and anti-obesity effects. Song *et al*. extracted the most effective RVS compound on adipogenesis, butein. Butein at a dosage of 10 μM strongly inhibited lipid accumulation in C3H10T1/2 and 3T3/L1 cells, and the effect was even stronger at higher doses. In addition, butein decreased the expression of PPARγ, aP2, and LPL mRNA in C3H10Tt1/2 cells. STAT3 is one of the transcriptional cascades in which PPARγ is involved. The data from Song *et al*. showed that butein regulates STAT3 in the early stages of adipocyte differentiation. Taken together, these data suggest that the anti-adipogenic effects of butein are achieved, at least in part, by suppressing STAT3 activation (84). *Rhizome polygonati falcatum* (RPF) is a medicinal plant in Japan, China, and Korea. RPF has pharmacological properties such as anti-hyperglycemia, anti-hyperlipidemia, and anti-atherosclerosis effects. Park *et al*. studied the impact of RPF extract and its component kaemferol, a natural flavonoid, during adipogenesis. 3T3-L1 cells treated with 0.5 mg/ml of RPF extract and 40 μM of kaempferol showed a significant reduction in lipid accumulation and TG content. RT-qPCR revealed that both treatments decreased the mRNA level of SREBP-1c and PPARγ and its target genes, adipcin and LPL. Consistently, kaempferol treatment decreased the expression of genes involved in fatty acid uptake and transport, such as Lpl, aP2, and CD36. In contrast, lipolysis-associated genes were up-regulated, inhibiting adipogenesis (85). *Salix pseudo-lasiogyne* twigs from the Salicaceae family have anti-inflammatory, anti-tumor, anti-oxidant, and anti-obesity properties and have traditionally been used to treat inflammation, pain, and fever. Lee *et al*. extracted five salicorin derivatives from an EtOAc fraction of the plant, with 2’,6’-O-acetylsalicortin (15) showing the strongest inhibitory activity on adipocyte development at 25 and 50 μM concentrations. Treatment with 1 significantly reduced C/EBPα regulation and gene expression in preadipocytes, including C/EBPβ and C/EBPδ. Furthermore, treatment with 1 reduced the gene and protein expression levels of SREBP-1c in a dose-dependent manner, as well as its target genes SCD-1, FAS, and ACC. Lee *et al*.’s findings implied that 1 inhibited adipocyte development through lipogenesis regulation (15). Wang *et al*. tested the effect of desmethylicaritin, a bioactive metabolite of *Epimedium* flavonoids in serum, on adipogenesis *in vitro*. Desmethylicaritin in 0.1, 1, and 10 µM decreased the lipid droplets of 3T3-L1 cells. Also, 10 µM of desmethylicaritin reduced the clonal expansion compared to the induced group. Treatment with desmethylicaritin significantly down-regulated the mRNA expression of C/EBPα and PPARγ. Consistently, it decreased the mRNA expression of FABP and LPL. In addition, desmethylicaritin up-regulated Wnt10b and was able to promote nuclear translocation of β-cantinin, which might play an essential role in suppressing adipogenesis (86). Guo *et al*. investigated the effects of *p*-synephrine on the differentiation of adipogenesis. *p*-synephrine is the primary protoalkaloid from *Citrus aurantium*, and it is often used as an herbal or dietary supplement in many countries worldwide. Treatment with *p*-synefhrine at 1 and 10 µM for six days significantly reduced lipid accumulation and TG content. Consistently, mRNA and protein expression of PPARγ, C/EBPα, and aP2 were decreased after treatment with *p*-synephrine. The compound also affected Akt/GSK3β pathway by decreasing the phosphorylated forms of GS but augmenting the phosphorylation of Akt and GSK3β, suggesting that *p*-synephrine activated Akt and reduced GSK3β activity (87). *Chrysanthemum indicum* has been used as a traditional herbal tea and drink since ancient times. It is a yellow flowering plant and contains flavonoids, terpenoids, and phenolic compounds. Kim *et al*. investigated the anti-adipogenic mechanisms of the aqueous extract of *C. indicum *(CAE) in 3T3-L1 preadipocytes. MDI-induced accumulation of intracellular lipid and triglyceride was significantly reduced in CAE-treated differentiated cells. CAE treatment significantly down-regulated the expression of PPARγ, C/EBPα, FABP4, FAS, SCD-1, and perilipin. CAE at a 2 mg/ml dose inhibited the cell cycle transition from G0/G1 to S phase, which MDI promoted. The expression of cyclin D1, cyclin A1, and CDK2, which play a central role in cell cycle progression, was suppressed by CAE treatment. Moreover, CAE mitigated the phosphorylation of STAT3 and the expression of C/EBPβ. Akt, MEK-1, and ERK1/2 were attenuated by CAE treatment in a dose-dependent manner, showing its possible mechanisms on adipogenesis (88). Petasites japonicas is an edible and medicinal plant in Asian countries, including China, Japan, and Korea. S-petasin is a bioactive compound found in p. japonicas. Guo *et al*. showed that treatment with 0.31, 0.62, and 1.55 µM of s-petasin inhibited glucose uptake in a dose-dependent manner. It also decreased TG accumulation and PPARγ expression. Besides, s-petasin inhibited PPARγ target genes A-FABP, FAS, LPL, and CD36 (89). Myricetin (3, 5, 7, 3’,4’,5’-hexahydroxyflavone; Myr) is a naturally occurring flavonol widely distributed in fruits, vegetables, and medicinal plants. According to the study by Wang *et al*., myricetin effectively inhibited adipocyte differentiation and decreased TG content at concentrations of 50 and 100 µmol/L without affecting cell viability. The mRNA expression levels of C/EBPβ and δ were slightly suppressed in Myr-treated cells. Myr suppressed the expression of PPARγ and C/EBPα as well as SREBP-1c, especially at late stages of differentiation. Myr also down-regulated the mRNA expression of PGC-1, aP2, and GLUT4. The results showed that Myr treatment suppressed MAPK signaling during preadipocyte differentiation but activated MAPK during adipocyte lipolysis and enhanced the phosphorylation of ERK, JNK, and p38, which promoted lipolysis in 3T3-L1 adipocytes (90). 

Raspberry ketone [4-(4-hydroxyphenyl) buran-2-one; RK] is a natural phenolic compound often used as a fragrance in cosmetics and as a flavoring agent in food. In a study, Park investigated a possible mechanism for the anti-obesity effect of RK. The result showed that treatment of preadipocytes with RK at a concentration of 10 µM and above reduced lipid accumulation. It also suppressed the expression of C/EBPα, PPARγ, and aP2. In addition, mRNA transcription of ACC, FAS, and SCD1 was down-regulated by RK. RK treatment significantly increased the expression of ATGL and HSL, two important lipases, and CPT1B. This oxidative enzyme enables adipose tissue to take up fatty acids from circulating triacylgelycerol, demonstrating the anti-obesity mechanism of RK (91). *Zanthoxylum schinifolium* is widespread throughout East Asia and is used as a food flavoring. In a study, Choi *et al*. tested the effect of 50, 100, 150, and 250 µg/ml ethanol extract from *Z. schinifolium* (EEZS) leaves on the differentiation of adipocytes. Microscopic observations showed that the number of lipid droplets and TG levels decreased with increasing EEZS concentration. In addition, EEZS treatment suppressed the expression of PPARγ, C/EBPα, and C/EBPβ compared to the fully differentiated control adipocytes. Treatment with 200 µg/ml EEZS effectively suppressed the phosphorylation of ERK, the levels of phosphorylated PI3K and Akt, suggesting that the inhibition of adipocyte differentiation by EEZS was associated with the inactivation of ERK and PI3K/Akt signaling pathway (92). *Oroxylum indicum* is a medicinal plant with major constituents of baicalein, baicalin, oroxylin A, and chrysin. Singh and Kakkar selected oroxylin A (OA) from this plant for its apoptotic activity on cancer cells. Treatment with 10, 20, and 40 µM of OA decreased the number of lipid droplets and accumulation in a dose-dependent manner, particularly from day 0 to day 2 of differentiation. Cells treated with 20 µM of OA also exhibited reduced PPARγ, FAS, and LPL levels. Results indicated that the release of free glycerol was significantly higher in OA-treated adipocytes on day 10. Furthermore, treatment with 40 µM of OA resulted in a significant number of mature adipocytes undergoing apoptosis. These findings strongly suggested the involvement of apoptosis in adipocytes in response to OA treatment (93). 

*Persicaria hydropiper* (L.) Spash is an edible wild herb grown worldwide in temperate climates. Lee *et al*. studied the impact of *P. hydropiper* and its flavonoid components, isoquercitrin, and isorhamnetin, on the activation of the Wnt/β-catenin pathway during adipocyte differentiation. The results showed that treatment with 1 µg/ml of *P. hydropiper* extract specifically activated the Wnt/β-catenin signaling pathway. It was observed that 50 µM of isoquercitrin and isorhamnetin increased the Wnt/β-catenin signaling pathway. However, the degree of activation was halved by isorhamnetin (17). 2, 4, 5-Trimethoxybenzaldehyde (TMBA) is a bitter principle found in plant roots, seeds, and leaves. Wu *et al*. isolated 2, 4, 5-TMBA from carrot (*Daucus carota* L.) seeds and cultured it with preadipocytes at 100 µg/ml concentration during differentiation. Co-culturing with 2, 4, 5-TMBA significantly suppressed the expression of phosphorylated MEK and ERK1, which are required to differentiate preadipocytes. In the culture study, C/EBPα, C/EBPβ, C/EBPδ, and PPARγ1 were down-regulated. The expression of ADD1 was also attenuated by 2, 4, 5-TMBA. Treatment with 2, 4, 5-TMBA significantly inhibited the expression of COX-2, perilipin A, and ACC during differentiation. Results showed that 100 µg/ml of 2, 4, 5-TMBA decreased the amount of lipid accumulation in fully differentiated adipocytes and increased glycerol release, indicating the inducing effect of 2, 4, 5-TMBA on lipolysis. The expression of perilipin A was suppressed, and HSL was up-regulated in mature adipocytes. Taken together, 2, 4, 5-TMBA suppressed adipogenesis and promoted lipolysis in 3T3-L1 adipocytes (94). To determine the antiadipogenic effect, Lee *et al*. investigated 1β-hydroxy-2-oxopomolic acid (HOA) isolated from *Agrimonia pilosa*, a perennial herbaceous flowering plant. The result showed that cells treated with 100 µM HOA reduced lipid accumulation. PPARγ and C/EBPα mRNA expression and protein levels were also reduced. Treatment with 50 and 100 µM HOA effectively reduced the mRNA expression of ADD1, SREBP1c, and resistin. GLUT4 mRNA expression was significantly reduced by HOA treatment after 8 days. HOA at higher doses (100 µM) also suppressed the expression of aP2, adiponectin, FAS, and resistin. These results suggested that HOA inhibits adipocyte differentiation by down-regulating adipogenic genes (95). Kim *et al*. studied the inhibitory effect of a butanol-soluble fraction (SPB) and isolated compounds from *Spirodela polyrhiza* ethanol extract on adipogenesis. SPB at concentrations of 20, 40, 100, and 200 µg/ml decreased triglyceride (TG) accumulation in a dose-dependent manner without causing cytotoxicity. Additionally, SPB inhibited the expression of PPARγ and C/EBPα. Kim *et al*. identified vitexin and orientin, flavonoid compounds, in SPB. Treatment with vitexin and orientin at a concentration of 100 µM decreased C/EBPα and PPARγ protein expression levels in 3T3-L1 cells, respectively (96). *Alnus hirsuta* f. *sibirica* is an indigenous *Alnus* species found in Korea. The bark of this plant has been used as an antipyretic and in health tea for alcoholism. Lee *et al*. extracted 18 diarylheptanoids from *A. hirsuta* f. *sibirica* leaves. Among all 18 diarylheptanoids, compound 7 showed the most effective activity on adipogenesis. Treatment with compound 7 at a concentration of 100 µM inhibited lipid accumulation and decreased PPARγ protein expression (97). Choi *et al*. investigated the effects of methanol extract from the rhizome of *Coptis chinensis* on adipocyte differentiation of 3T3-L1 cells. Rhizome of *Coptis chinensis* Franch. has been shown to have antidiabetic, antifungal, antimicrobial, and antiviral effects. Choi and his group isolated five alkaloids, berberine, epiberberine, coptisine, palmatine, and magnoflorine, from the Coptidis rhizoma. The MeOH extract of Coptidis rhizoma at a concentration of 12.5, 25, and 50 µg/ml inhibited cellular TG accumulation in a dose-dependent manner. 3T3-L1 cells were treated with all five alkaloids at 12.5, 25, and 50 µg/ml doses. Berberine, epiberberine, and coptisine showed the strongest inhibitory effect on lipid accumulation. Palmatine showed only weak inhibition, and treatment with magnoflorine resulted in a moderate reduction of lipid accumulation in 3T3-L1 adipocytes. In addition, all five compounds suppressed the expression of PPARγ and C/EBPα compared to control adipocytes (20). In another study, Choi *et al*. investigated the molecular mechanism of epiberberine in particular. Treatment with epiberberine at concentrations of 12.5, 25, and 50 µM reduced lipid accumulation in a dose-dependent fashion. It also suppressed the expression levels of SREBP-1c and FAS. Administration of epiberberine inhibited the phosphorylation of ERK1/2 and its upstream signaling molecules, such as p44/42, c-Raf, and MEK1/2. Moreover, epiberberine suppressed the phosphorylation of AMPKα and Akt (98). *Aspalathus linearis *(rooibos) is a South African herbal tea with a rich complement of polyphenols, including flavonoids. It demonstrated anti-oxidant, antimutagenic, and anticancer effects. Sanderson *et al*. studied hot water soluble solids (HWSS) extracted from fermented rooibos when preparing an infusion at a “cup-of-tea” on adipocyte development. Treatment with 10 and 100 µg/ml of the rooibos HWSS appeared to inhibit lipid accumulation. Rooibos HWSS decreased the mRNA abundance of PPARγ, PPARα, SREBP1c, and FASN at a 100 µg/ml concentration, respectively. Chronic administration of the rooibos HWSS enhanced cellular metabolism by elevating the ATP content in differentiating 3T3-L1 adipocytes. It also significantly decreased leptin secretion, indicating that chronic rooibos consumption might inhibit adipogenesis (99). Beg *et al*. evaluated the abundant and most effective withanolide of *Withania coagulans*, coagulin-L, for its effects on adipogenesis. *W. coagulans* has been used for the treatment of type 2 diabetes. It exhibits hepatoprotective and hypolipidemic activities as well (100). The addition of coagulin-L decreased the accumulation of lipid droplets. Microscopic observations revealed that coagulin-L decreased PPARγ, C/EBPα, and GLUT4 levels on days 2, 4, and 6. Importantly, the expression of aP2, LPL, FAS, and SREBP-1c was down-regulated upon the addition of coagulin-L. Cells treated with coagulin-L for days 0–2, 0–4, and 0–6 showed highly significant inhibition, suggesting that early exposure was necessary to suppress lipid accumulation. Treatment with coagulin-L prevented the reduction of the expression levels of the Wnt/β-catenin pathway and mediated the inhibition of adipogenesis (100). Cranberries (*Oxycoccus quadripetalus*) are a valuable source of bioactive substances rich in vitamins, microelements, and polyphenols with beneficial health properties. Kowalska *et al*. presented results of *in vitro* studies regarding the effects of cranberries on adipocytes. Treatment with cranberries at doses of 5 to 20 mg/ml decreased the fat content. Cranberries at the highest dose of 20 mg/ml caused a reduction in the ROS level compared to the control adipocytes. Analysis of the effect of cranberries showed that it stimulated lipolysis in the 3T3-L1 mature adipocytes. The treatment of differentiated adipocytes with 20 mg/ml of cranberries elevated the glycerol release from adipocytes. These results showed that cranberries may act directly on adipocytes to inhibit lipogenesis and stimulate lipolysis. The results indicated that cranberries suppressed the expression of PPARγ, C/EBPα, and SREBP-1c at concentrations of 2.5, 5, and 10 mg/ml (101). 

*Daphne genkwai* Siebold et Zuccarini (GFF) is a Chinese herbal remedy mainly used in China and Korea. Apigenin is a naturally occurring plant flavone found in common fruits and vegetables. Kim *et al*. extracted apigenin from the dried flowers of *D. genkwa* and investigated the role of apigenin in adipogenic differentiation of 3T3-L1 cells. The results showed that GFF at 25, 50, and 75 µg/ml concentrations and apigenin at 70 µM reduced lipid accumulation without cytotoxic effects. When the cells were treated with apigenin in the early differentiation phase (day 0 to day 2), the formation of lipid droplets was significantly reduced. The results show that treatment of preadipocytes with apigenin blocks the MCE phase of differentiation. Apigenin also down-regulated the expression of early adipogenic transcription factors, C/EBPβ, C/EBPα, and PPARγ (102). Lee *et al*. studied the effects of kaempferol, a compound from *Nelumbo nucifera* Gaertn, on adipogenesis. Their results showed that kaempferol treatment (2.5–40 µM) significantly reduced cellular TG content. Kaempferol broadly inhibited the expression levels of C/EBPα, PPARγ, RXRα, LXRα, and SREBP-1c. Additionally, the mRNA and protein levels of FAS, SCD1, and ACCα were reduced in a dose-dependent manner (103). 

SIRT1 is a critical factor that controls adipocyte fat storage and glucose homeostasis. Wang *et al*. indicated that 3T3-L1 adipocytes were treated with 3 and 10 µM of agrimol B, a polyphenol derived from *Agrimonia pilosa *Ladeb. significantly induced cytoplasm-to-nucleus shuttle of SIRT1. It also dramatically reduced PPARγ, C/EBPα, FAS, UCP-1, and apoE expression, balancing obesity (104). Cis-3’,4’-diisovalerylkhellactone (cDIVK) is a khellactone coumarin isolated from the leaves of *Peucedanum japonicum* Thunb. Choi *et al*.’s study showed that 30 and 50 µM of cDIVK effectively inhibited adipocyte differentiation. cDIVK treatment suppressed the mRNA and protein expression of PPARγ, C/EBPα, and SREBP-1c. 30 and 50 µM of cDIVK significantly increased glucose uptake into the adipocytes compared with the control. It also potentially inhibited α-glucosidase, showing its anti-obesity and antidiabetic activity (105). The study by Song *et al*. on the leaves of the plant *Dendropanax morbiferus*, a beneficial plant rich in vitamin C and tannin, showed that the extract from the water of *D. morbiferus* (DLW) at a concentration of 5–500 µg/ml caused a strong reduction in cellular TG levels and glucose uptake. In addition, the mRNA and protein levels of C/EBPα, C/EBPβ, and PPARγ were decreased by DLW, as well as the protein expression of SREBP-1c and FAS (106). *Boussingaultia gracilis* Miers var. *pseudobaselloides *Bailey fresh leaves are often used as a vegetable and folk medicine. Kim and Choung found that the ethanol extract of *B. gracilis* Miers var. *pseudobaselloides* Bailey (BGE) affected adipocyte differentiation mainly by increasing the levels of phosphorylated AMPK and ACC at 10, 50, and 100 µg/ml compared to control groups. BGE treatment also decreased PPARγ and C/EBPα expression, even at the lowest concentration. At the same time, the SREBP-1c gene and its target gene, FAS, were down-regulated in BGE-treated 3T3-L1 cells (107). Mulberry contains phenolic compounds and has been used as a traditional oriental medicine for a long time. Yang *et al*. reported that treatment with mulberry leaf ethanol extract (MLEE) for 8 days at concentrations of 10, 25, 50, and 100 µg/ml significantly reduced protein levels of PPARγ, FAS, adiponectin, and PGC-1α, which stimulate mitochondrial biogenesis and adaptive thermogenesis, in differentiated adipocytes (108). Edible flowers of *Tropaeolum majus* have been used as a disinfectant and antibiotic for wound healing. Kim *et al*. showed that *T. majus* ethanol extract (TME) at concentrations of 20, 300, and 500 µg/ml reduced lipid accumulation, with the lowest accumulation rate observed in cells treated with 300 µg/ml TME. Treatment with TME resulted in a decrease in PPARγ, C/EBPα, and SREBP-1c in a concentration-dependent manner (109). *Cornus kousa*, a Korean dogwood, has been traditionally used as therapeutic medicine in East Asia. Imran Khan *et al*. extracted dried plant leaves with ethanol and obtained an anthocyanin fraction (Ant Fr). Their results showed that Ant Fr suppressed angiogenesis by inhibiting proliferation and down-regulating VEGFR2, PI3K, β-catenin, NF-κB, and Akt1 in a dose-dependent manner from 5 to 100 µg/ml. Ant Fr also inhibited lipid accumulation by reducing the expression of PPARγ, CCAAT, C/EBPα, aP2, FAS, and LPL and enhancing the activation of the AMPK signaling pathway, demonstrating a positive impact on treating or controlling obesity (110). Like the previous study, Bu *et al*. showed that bilobalide, the only sesquiterpene from *Ginkgo biloba* leaf, suppressed adipogenesis via the AMPK signaling pathway. Treatment with 25 and 100 µM of bilobalide significantly enhanced the phosphorylation of AMPK and ACC1. Additionally, bilobalide treatment promoted the expression of genes involved in lipolysis, including ATGL, HSL, and CPT1α. Moreover, treatment with bilobalide from day 4 to day 8 decreased the mRNA and protein expression of PPARγ, C/EBPα, and SREBP-1c in a concentration-dependent manner (111). *Mesona procumbens* is a common ingredient used to prepare functional beverages in many Asian countries. Huang *et al*. isolated eight primeverose derivatives, mesonosides A-H, from the methanolic extract of *M. procumbens*. The results revealed that all compounds inhibited lipid accumulation by reducing the protein levels of PPARγ and C/EBPα after 8 days of treatment. Notably, compound 3 exhibited the most inhibition of lipid accumulation and protein levels of PPARγ and C/EBPα (112). Zarasvand *et al*.’s study showed that mangiferin (MGF) and mango leaf tea (MLT) from *Mangifera indica* L. at a concentration of 100 µM both increased glucose uptake. However, only MLT decreased TG accumulation in adipocytes. MLT treatment significantly increased the mRNA expression levels of FOXO1 and ATGL. In contrast, ACC was decreased in MLT- and MGF-treated cells. These results indicated the regulatory ability of MLT on adipogenesis by altering glucose/insulin-responsive genes in adipocytes (113). *Zingiber officinale *Roscoe, or ginger, is a medicinal plant with an anti-obesogenic effect mostly through its gingerols. Gembe-olivarez *et al*. evaluated the lipolytic and anti-adipogenic activity of a mixture of the main ginger phenols: 6-gingerol, 8-gingerol, 10-gingerol, 6-shogaol, 8-shogaol, and 10-shogaol on the 3T3-L1 cell line. The findings indicated that treatment with 2 µg/ml of the phenol mix during and after adipogenesis decreased lipid content. Mature adipocytes treated with the phenol mix presented a markedly lower C/EBPα, FABP4, and FASN expression than non-differentiated and positive control groups (114). 

## Anti-adipogenic effects of phytochemicals and herbs in human cell lines

Several pharmacological studies have demonstrated that *Ononis spinosa* exhibited antimicrobial, anti-oxidant, anti-inflammatory, diuretic, analgesic, anticancer, dermatological, and hepatoprotective properties (115). In a study, the anti-adipogenic effects of *Ononis spinosa* L. roots and its secondary metabolites ononin and maackiain in human preadipocyte cell strain Simpson-Golabi-Behmel syndrome as an *in vitro* model of obesity was observed. Maackiain (5, 10, 25, and 50 μM) for 24 hr significantly reduced lipid accumulation, indicating a potent antiadipogenic effect. The mechanism of action involves modulation of PI3K, PPARγ, and C/EBPα signaling pathways. It also restricted lipogenesis by inhibiting CEBPA, AKT, SREBP1, ACC, and ADIPOQ. Like maackiain, pure ononin (5, 10, and 25 μM) for 24 hr also significantly reduced lipid accumulation. The most likely mechanism of action is the suppression of PI3K and PPARγ, activation of SIRT1, and subsequent inhibition of PPAR/adiponectin axis activity. The results showed that the inhibitory effect of adipolysis by ononin was higher in human adipocytes (116). Similarly, the San *et al*. study showed that pinostrobin at 5-20 µM for 48 hr significantly reduced intracellular lipid accumulation in human (PCS-210-010) preadipocytes (21). In addition, Spalletta *et al*. showed that carvacrol at 25 μM decreased cell differentiation in WJ-1 and WJ-2 (Wharton’s jelly-derived mesenchymal stem cells) cell lines. They found that carvacrol did not appear to impair autophagy effectively in WJ-1/2 cells compared to 3T3-L1 cells, as shown by the analysis of LC3-II levels. However, ultrastructural analysis showed that carvacrol treatment decreased the formation of autophagic bodies during adipogenic differentiation in WJ-1 cells, suggesting that carvacrol may affect autophagy through mechanisms not directly reflected in LC3-II levels (16). Resveratrol (3, 4’,5-trans- trihydroxystilbene) and piceatannol (3,3’,4’5-trans-tetraphydroxy-stilbene) are particular stilbene compounds that can be found in natural foods, such as berries and fruits. Park *et al*. reported that at 5, 10, and 20 µM, both compounds were effective for inhibiting adipogenesis of vASCs. However, the reduction in mRNA expression of C/EBPα, PPARγ, and aP2 at 20 µM of piceatannol was higher than in the resveratrol-treated group, indicating that piceatennol is more effective (117). Muscadine grape is the native grape species in the Southern states, and its nutraceutical benefits have been well known. Zhao *et al*. studied muscadine grape seed oil (MGSO) as a novel source of tocotrinols that are an unsaturated form of vitamin E in human adipose-derived stem cells (hASCs). It was found that 200 µM MGSO and 5.7 µg/ml MGSO-derived tocotrienol reduced the mRNA expression of PPARγ and CEBPα, which are crucial to adipogenesis. In addition, tocotrienol showed a stronger outcome in inhibiting the mRNA expression of other adipocyte genes such as aP2, FAS, and perilipin and protein expression of CEBPα, aP2, and FAS (118). Nerurkar and colleagues conducted another study on primary human preadipocytes. Their targeted plant, *Momordica charantia*, known as bitter melon, is widely grown in Asia, East- Africa, and South America and is greatly used as folk medicine. A significant reduction in SREBP-1c, PPARγ, and resistin mRNA expression was observed with 2% bitter melon juice (v/v) treatment (119). The juice of *Citrus bergamia *is a rich source of flavonoids, such as naringin, hesperidin, and neohesperidin, with a major role in human health. Administration of 10 or 100 µg/ml of *C. bergamia *juice to mesenchymal stem cells from human adipose tissue decreased the levels of PPARγ and A-FABP, a cytoplasmic protein highly expressed in adipocytes. Also, the production of lipolysis markers, ATGL, HSL, and MGL was up-regulated (120). Park *et al*. investigated coumarin compounds, decursin (D) and decursinol angelate (DA), present in *Angelica gigas *Nakai, known as ’Korean dang-gui in human visceral adipose-derived stem cells. The protein expression of PPARγ, C/EBPα, and aP2 reduced after administration of 40 μM D and DA. The mRNA levels of lipogenic markers, FAS, ACC, and SREBP-1c were also decreased. D and DA increased expression and the nuclear translocation of β-catenin (121). Nehme *et al*. showed a substantial decrease in leptin gene expression after human monocytic leukemia cells were treated with rooibos and an increase in the adiponectin gene. Rooibos greatly enhanced the expression of adipoQ and HSL compared to the control group (122). Jafari *et al.* reported that Saffron, crocin, and crocetin significantly inhibited adipocyte differentiation by reducing PPARγ, GAPDH, and FAS protein levels (123). Also, Shahbodi *et al.* revealed that thymoquinone reduced the differentiation of fat stem cells into fat cells by suppressing the expression of PPARγ and FAS proteins (124). Another study presents a variety of natural products with different mechanisms of action targeting different stages of adipogenesis. These mechanisms include modulation of key signaling pathways such as PI3K, PPAR, C/EBPα, SREBP1, ACC, AMPK, and others. This diversity suggests that these compounds may have a multifaceted impact on adipogenesis. Adipogenesis is a complex process regulated by transcriptional factors such as SREBP1, C/EBPα, and PPARγ. These transcription factors controlled the lipid homeostasis by modifying the expression of target genes FAS, FABP4, leptin, and AP2. On the other hand, activation of AMPK, as a major regulator of cellular energy balance, resulted in suppression of SREBP1, PPARγ, and C/EBPα, as well as inhibition of ACC activity (125). Furthermore, MAPKs and Wnt/β-catenin signaling pathways (JNK, P38, and ERK1/2) can attenuate PPARγ transactivation function via inhibitory phosphorylation (126). In addition, PI3K/Akt, PKA activation markedly enhanced CREB phosphorylation and transcriptional activity of PPAR-γ (127). As we have seen in the text, cell models such as 3T3-L1 preadipocytes and human preadipocytes are commonly used in the above studies. While these models provide valuable insights into potential mechanisms, it is important to recognize that the results of cell-based studies do not always translate directly to clinical outcomes. This is the first study that summarizes natural products in one article and elaborates on their possible mechanisms of adipogenesis (Figure 1).

Nevertheless, our study has some limitations. First, we cannot access some full texts, which could affect our judgment. Second, concentration and duration were different in each study. Third, each compound mentioned in the text has specific effects on different stages of adipogenesis and related signaling pathways, which we considered a limitation. Since the effectiveness of phytochemicals on human health is still in question, more clinical studies on humans are needed to gain in-depth insight into the anti-adipogenic properties of different phytochemicals and herbs. Thus, more clinical and animal studies are needed on probable molecular mechanisms of phytochemicals. Finally, most studies were unclear in assessing the risk of bias; they included a detailed explanation of the methodology, randomization, method of measuring the results, and other sources of bias. This diversity of sources and the large number of studies could affect the derivation of results.

The following article provides the reader with comprehensive information on the action of plants and phytochemicals as suppressors of adipogenesis. Translating laboratory results into clinical applications can be challenging due to various factors such as bioavailability, dosing, and potential interactions with other drugs. Researchers must address these challenges to ensure a successful transition from the laboratory to the bedside. Finally, explaining that animal studies and clinical trials are necessary to reject or confirm the available evidence is important. Focusing on this area may lead to good results in controlling and treating obesity and related diseases.

**Table 1:** Overview of the PICO (Participants intervention comparator outcomes) eligibility criteria

Participants	Cell lines subjected to phytochemicals There will be no restriction on the type of cell line and doses of phytochemicals
Intervention	Phytochemicals that have anti-adipogenesis ability
Comparator	Comparison will be between control and experiment intervention. Studies without a control group will be excluded
Outcomes	Our primary outcome is the inhibition of adipogenesis and lowering of lipid accumulation. Studies without inhibitory effects on adipogenesis will be excluded. Our secondary outcome is significant depletion of factors and/or enzymes involved in adipogenesis
Study design	Studies will be limited to experimental prospective controlled studies. Non-comparative studies, systematic reviews and meta-analyses, *in vivo* studies, and opinion and editorials will be excluded

## Conclusion

In summary, *in vitro* studies have demonstrated the potential of phytochemicals as effective inhibitors of adipogenesis. These bioactive compounds interfere with key adipogenic transcription factors, modulate signaling pathways, and exhibit anti-inflammatory properties that collectively lead to reduced lipid accumulation and adipocyte differentiation. Harnessing the therapeutic potential of phytochemicals may offer novel and sustainable approaches to combat obesity and its associated health risks. As we continue to unravel the complexity of the effect of phytochemicals on adipogenesis, further investigation, and translational research may pave the way for the development of phytochemical-based interventions to treat obesity and improve metabolic health.

## Declaration

We have not used any AI tools or technologies to prepare this manuscript.
