# Supplementary material for: In vitro effects of phytochemicals on adipogenesis with a focus on molecular mechanisms: A systematic review
Source: Iran J Basic Med Sci. 2025;28(4):409–25. doi: 10.22038/ijbms.2025.78924.17090 (PMC11831750; doi:10.22038/ijbms.2025.78924.17090)
Supplement: Supplementary file 1 [file ijbms-28-409-s001.pdf]

Table 2. Detailed information about the *in vitro* studies of phytochemicals on adipogenesis and the related molecular mechanism

Cell line	Intervention	Concentration/ duration	Results intervention/ outcomes	Ref.
3T3-L1 mouse preadipocyte	Hypersampsone P (*Hypericum subsessile*)	5-25 µM, 7 days	↓expressions of PPARγ and FABP4	(6)
3T3-L1 preadipocyte	Aster yomena	200 µg/ml, 7 days	↓expression of C/EBP-α&β, PPAR-γ, SREBP-1c, ↑ AMPK	(11)
3T3-L1 mouse embryo fibroblast	Esculetin	0 to 800 µM, up to 48 hr and 6 days	↑adipocyte apoptosis, ↓adipocyte differentiation occurred during the early, intermediate, and late stages	(12)
3T3-L1 preadipocyte	*Salix pseudo-lasiogyne*	25, 50 µM, 8 days	↓expressions of C/EBPα and SREBP1c, suppressed mRNA expression levels of C/EBPβ, SCCD-1, ACC and FAS	(13)
Mouse embryo fibroblast 3T3-L1 cell	Salicortin-Derivatives (*Salix pseudo-lasiogyne*)	25, 50 µM 8 days	↓expressions of C/EBPα and SREBP1c, suppressed mRNA expression levels of C/EBPβ, SCD1, ACC, FAS	(13)
murine 3T3-L1 human WJ-MSC	Carvacrol	25 µM, 7 and 17 days	↓cell differentiation ↓autophagy, ↓ChREBP expression ↓ formation of autophagic bodies	(14)
3T3-L1 preadipocyte	*Persicaria hydropiper* (L.)	1 µg/mL, 5 µg/mL 7 days	↑activation of Wnt/β-catenin signaling pathway	(15)
3T3-L1 preadipocyte	Carnosic acid (*Rosmarinus officinalis*)	10, 20, 30 µg/ml 6 days	↓expression of PPARγ and FABP4 and alteration the subnuclear distribution of C/EBPβ ↑LIP/LAP ratio	(16)
3T3-L1 preadipocyte	*Coptis chinensis*	12.5–50 µM, 8 days	↓triglyceride content ↓expression and protein levels of C/EBPα and PPAR	(18)
mouse 3T3-L1 pre-adipocyte	Epiberberine (*Coptis chinensis*)	12.5, 25, or 50 µM Up to 8 days	↓SREBP-1 suppressed the differentiation-mediated phosphorylation of MEK1/ERK1/2 and AMPKα/Akt pathways ↓gene expression of FAS	(18)
mouse (3T3-L1) PCS-210-010	Pinostrobin (*Boesenbergia rotunda*)	5-20 µM,48 hr	↓C/EBP, PPARγ, SREBP-1c, and TG levels ↓p-Akt/Akt and p-GSK3β/GSK3β levels and ↑p-AMPKα/AMPKα and p-ACC/ACC levels	(19)
3T3-L1 preadipocyte	*Abeliophyllum distichum* leaf extract	50-200 µg/ml, 8 days	↓PPARγ, C/EBPα, LPL, ap2, ACC, FAS and phosphorylation of MAPK	(20)
3T3-L1 preadipocyte	gymnemic acids (*Gymnema inodorum*)	25-100 µg/ml, 10 days	↓ expression of Lipin-1, PPARγ, C/EBPα, FASN, CD36 and fatty acid binding protein 4 (FABP4) levels	(22)

3T3-L1 preadipocyte	Anthocyanin (*Vitis coignetiae Pulliat*)	50, 100, and μg/ml 8 days	↑expression of UCP1 and Pgc1α<br>↓lipid droplets, lipid content, and triglyceride production,<br>↑activation of AMPK and ACC,<br>↓expression of C/EBP-α&β, PPAR-γ, SREBP-1c, ↓aP2, leptin and FAS	(23)
3T3-L1 preadipocyte	Euphorbiasteroid (*Euphorbia lathyris* L.)	12.5, 25 and 50 μM, 8 days	↑phosphorylation of AMPK and ACC,<br>↓levels of adipogenic proteins PPARγ, C/EBPα and FAS,	(24)
3T3-L1 preadipocyte	Antofine (*Cynanchum paniculatum Kitagawa*)	10 nm, 48 and 96 hr	↓expression of PPARγ and AP2 levels	(25)
3T3-L1 mouse preadipocyte	*Cyclopia maculate* and *Cyclopia subternata*	20-1600 μg/ml, 8 days	↓intracellular TG and fat accumulation,<br>↓PPAR2 expression,<br>↓mitochondrial dehydrogenase activity,	(26)
3T3-L1 preadipocyte	*Sibiraea angustata*	50, 100 and 200 mg/Ml 7 days	↓ expression of PPAR, C/EBPβ, aP2, LPL and GLUT4,<br>blocked the G1–S transition phase	(27)
Mouse adipocytic 3T3-L1 cell	Quercetin-3-O-(600-feruloyl)-b-D-galactopyranoside (*Psidium guajava*)	10 μm, 9 days	↓mRNA and protein expression of PPAR, C/EBPα	(28)
3T3-L1 preadipocyte	Isorhamnetin 3-O-β-D-glucopyranoside (*Salicornia Herbacea*)	20 μm, 6 days	↓ PPARγ, C/EBPα and differentiation- SREBP1 and adipogen-specific proteins FAS, GLUT 4, retinoid X receptor (RXR)α, and leptin levels<br>↑AMPK	(29)
3T3-L1 preadipocyte	(+)-episesamin (*Lindera obtusiloba*)	10 μm, 8 days	↓ expression of GLUT-4, PPAR, phosphorylation of ERK1/2 and β-catenin,<br>↑protein expression of iNOS,<br>anti-inflammatory activity	(30)
3T3-L1 preadipocyte	P-coumaric acid *Sasa quelpaertensis* Nakai	SQE: 125, 250, 500 μg/ml p-Coumaric acid: 12.5, 25, 50, 100 μM 8 days	SQE: ↓expression of C/EBPα, PPAR, SREBP-1c, aP2, FAS and adiponectin<br>↑phosphorylation of AMPK and ACC,<br>p-Coumaric acid:<br>↓expression of C/EBPα, PPAR, SREBP-1c	(31)
3T3-L1 pre-adipocytes	Glycoside St-C1 and Glycoside St-E2 (*Acanthopanax henryi* (Oliv.))	1 μg/mL, 6 days	↓C/EBPα, PPAR<br>↑AMPK	(32)
Murine 3T3-L1 preadipocyte	Fucosterol (*Ecklonia stolonifera*)	25 and 50 μM 8 days	↓lipid accumulation,<br>↓PI3K/Akt and ERK pathways<br>↓expression of PPAR, C/EBPα and SREBP-1,<br>↑SIRT1 expression,<br>↓ phosphorylation of FoxO1	(33)
3T3-L1 preadipocyte	Tartary buckwheat	100 μg/ml	↓lipid accumulation, triglyceride content,	(33)

	(*Fagopyrum tataricum*)	Up to 7 days	and GPDH activity,<br>↓mRNA levels of PPAR, CEBP-α, aP2, ACC, FAS, SCD-1,<br>↓mRNA levels of TNF-α, IL-6, MCP-1 and INOS, NO production	
Murine 3T3-L1 preadipocyte	Monascus ruber-fermented<br>*Fagopyrum esculentum*	50-800 µg/mL<br>6 days	↓ expression of PPAR, C/EBPα, aP2, FAS and leptin, cyclin-dependent kinase 2 and cyclin expression,<br>↑p21 and p27 expression→ cell cycle arrest at the G1/S phase	(34)
3T3-MBX pre-adipocyte	Zyflamend	200 µg/ml, 12 days	↓proliferation, inhibited lipid accumulation and expression of lipogenic genes, cell lipolysis and death<br>↑ 3T3-MBX pre-adipocytes.<br>↓ FASN, PCB, C/EBP, adiponectin, PPARγ expression<br>↓PKA and JNK pathways	(35)
3T3-L1 preadipocyte	Apigetrin	100 µM, 8 days	↓cell proliferation during MCE,<br>↓the mRNA levels of C/EBP-α, PPAR-γ, SREBP-1c and FAS, TNF-α and IL-6<br>↓H_2O_2-induced production of ROS	(36)
	Kaempferol-3-O-rutinoside<br>(*Solidago virgaurea*)	10 µg/ml,<br>8 days	↓PPARγ and C/EBPα<br>expression	(37)
3T3-L1 preadipocyte	Berberine	5 µM,<br>7 days	↓mRNA expressions of C/EBPα, PPARγ2, SREBP1c, and LPL	(38)
3T3-L1 mouse fibroblast	Coumestrol	20, 40 µM,<br>8 days	↓protein expression of PPARγ, C/EBPα, aP2, FAS,<br>↓Akt and GSK3β phosphorylation,<br>↑β-catenin, Wnt10b and LRP6	(39)
Mouse adipocytic 3T3-L1 cell	Apigenin	1, 10, 50 µM<br>8 days	↓ expression of HSL and MGL, ATGL levels<br>↑AMPK	(40)
3T3-L1 preadipocyte	Dohongsamul-tang	62.5, 125, 250 and 500 µg/Ml<br>7 days	↓accumulation of TG, leptin, adiponectin, resistin and PAI-1<br>↓PPAR , C/EBPα , FABP4	(42)
3T3-L1 preadipocyte	Trigonelline<br>(*Trigonella Foenum Graecum*)	75 and 100 µM, 10 days	↓mRNA expression of PPARγ and C/EBPα, adipogenin, adiponectin, resistin, leptin, and AP2, FAS, GLUT-4	(43)
3T3-L1 adipocyte	Soyasaponins Aa and Ab	25, 50,<br>and 100 µM, 8 days	↓expression of adiponectin, SREBP1-c, FAS, aP2 and resistin<br>↓expression of mRNA<br>and protein levels of PPARγ and C/EBPα	(44)
3T3-L1 preadipocyte	Parthenolide	1-8 µM,<br>6 days	↓ protein abundance of C/EBPα, PPARγ, and FABP4<br>↑activation of Nrf2/Keap1 signaling	(45)
3T3-L1 preadipocyte	7,8-Dihydroxyflavone	1, 10, 20 µM,<br>8 days	↓expression of C/EBP-α&β, PPAR-γ, aP2<br>↓intracellular ROS level, MAPK pathway (p38 and ERK)<br>↑expression of Mn-SOD, CAT, HO-1	(46)
3T3-L1 preadipocyte	*Orostachys japonicus*	10, 50, and 100 µg/ml	↓ROS generation and lipid accumulation,	(47)

		8 days	↑SOD-1 and SOD-2 protein,<br>↓mRNA and protein expression of NOX4 and G6PDH,<br>↓ mRNA and protein<br>levels of PPAR, C/EBPα, aP2	
Mouse 3T3-L1 cell	Mulberry<br>(*Morus alba* L.)	50, and 100 ng/mL<br>7 days	↓lipid and triglyceride accumulation and GPDH activity<br>↓mRNA expression of PPAR, C/EBPα, aP2	(49)
3T3-L1 preadipocyte	Centipede grass	10, 100 μg/ml<br>6 days	↓expression of C/EBPα, C/EBPβ, PPAR, aP2<br>↓phosphorylation levels of Akt and GSK3β	(50)
3T3-L1 preadipocyte	Buckwheat sprout<br>(*Fagopyrum esculentum* M.)	50 μg/mL,<br>8 days	↓adipocyte differentiation and ROS production, NOX4 and the NADPH-producing enzyme G6PDH<br>↓C/EBPα, PPAR γ and aP2	(51)
3T3-L1 preadipocyte	Mogrol<br>(*Siraitia grosvenorii*)	20 μM<br>8 days	↑AMPK phosphorylation<br>↓glycerol-3-phosphate dehydrogenase<br>↓C/EBPβ	(52)
3T3-L1 adipocyte	Baicalein	50 μM<br>6 days	↓PPARγ, C/EBPα, FAS, SCD<br>↓glucose uptake and lipid accumulation,<br>↓binding level of C/EBPα protein to the promoter region of the GLUT4 gene<br>↓phosphorylation of Akt	(53)
3T3-L1 mouse fibroblast cell	*Ecklonia cava*	50 μg/ml,<br>24 h	↓glucose utilization and TG accumulation<br>↓ expression of C/EBPα,<br>↓expression levels of SREBP-1c, A-FABP, FAS and adiponectin	(55)
3T3-L1 preadipocyte	Guarana (*Paullinia cupana*)	100, 150, 200 and 300 μg/mL<br>96 hr	↑Wnt10b, Wnt3a, Wnt1, Gata3, Dlk1, β -catenin nuclear translocation<br>↓C/EBPα, PPAR and CREB1<br>↓mmu-miR-27b-3p, mmu-miR-34b-5p and mmu-miR-760-5p	(56)
3T3-L1 preadipocyte and PWATs from C57BL/6 mice	Black Ginseng and Ginsenoside Rb1	BG: 25, 50,<br>and 100 μg/Ml<br>Rb1: 10, 20, and 40 μM<br>7 days	↓ expressions of C/EBPα and SREBP-1c,<br>↑protein expressions of PRDM16, PGC-1α and UCP1,<br>↑AMPK	(57)
3T3-L1 preadipocyte	Saikosaponin A (SSA) and Saikosaponin D<br>(SSD)<br>(*Bupleurum chinensis*)	0.938, 1.875, 3.75, 7.5 and 15 μM<br>8 days	↓expression of PPAR γ, C/EBPα, SREBP-1c and adiponectin, FABP4, FAS, LPL<br>↑phosphorylation of AMPK, ACC<br>↓phosphorylation of ERK1/2 and p38	(58)
3T3-L1 preadipocyte	6-gingerol	5, 10, 15 μg/ml<br>7 days	↓mRNA expression levels of PPAR-γ and C/EBP-α and the key lipogenic enzymes,<br>↑ Wnt/β-catenin signaling pathway	(59)
3T3-L1 mouse embryo fibroblast	*Edgeworthia gardneri* (wall.) Meisn.	12.5, 25, 50, 100 μg/ml<br>8 days	↓lipid and triglyceride accumulations,<br>↓PPAR and C/EBPα	(60)

			↑AMPK and ACC phosphorylation	
3T3-L1 preadipocyte	Andrographolide (*AndrographiPaniculata*)	7.5, 15 µM 8 days	↓C/EBPα, C/EBPβ mRNA and protein expression, PPAR protein level, FAS, stearoyl-CoA desaturase expression and lipid accumulation, ↓ phosphorylation of PKA ↓cyclin A, cyclin E, and CDK2 expression	(61)
3T3-L1 preadipocyte	Mansorins and mansonones (*Mansonia gagei*)	10 µM	↓level of aP2 and adiponectin, ↓expression of C/EBPα	(62)
murine 3T3L1 pre-adipocytes	Quercetin-3-O-rutinoside (*Moringa oleifera*)	20, 40, and 80 µg/ml 12 days	↓digestive enzymes ↑glucose uptake ↑mRNA levels of UCP-1 and GLUT-4 ↓PPAR-γ, adiponectin levels	(63)
3T3-L1 preadipocyte	*Ephedrae herba* and *Coicis semen*	25 µg/ml, 8 days	↓lipid accumulation ↑AMPK ↓PPAR, FABP4, and CEBP gene expression ↓SCD1, FASN, ACC1, and SREBF1 gene expression	(64)
3T3-L1 preadipocyte	Withanolide (*Withania somnifera*)	25 µM, 10 days	↓adipogeneesis and enlargement of lipid droplets, ↓FABP4 and Adipsin mRNA expression, ↑expression of the HSL and ATGL, ↓expression of the SREBP1	(65)
3T3-L1 preadipocyte	Syringin (*Cirsium brevicaule*)	2.5-200 µM, 48 hr	↓lipid accumulation ↓expressions of PPARγ, ↑phosphorylation of AMPK and ACC	(66)
3T3-L1 preadipocyte	BS21 (*Phyllostachys pubescens* leaves and *Scutellaria baicalensis* roots)	60-480 µg/ml, 7 days	↓expression of PPARγ, C/EBPα, aP2, SREBP-1c and FAS, ↑protein levels of CPT1 and p-ACC, induction of PRDM16, PGC1α, UCP1, and AMPK activation	(67)
3T3-L1 preadipocyte	HCF (*Chrysanthemum morifolium Ramat*)	0.1, 0.5, 1 µg/ml , 7 days	↓PPARγ, C/EBPα, SREBP-1c, FABP4, ACC1 and FAS, ↑AMPK and SIRT1 activation	(68)
3T3-L1 preadipocyte	Gypsogenin (*Momordica cochinchinensis*)	25-100 µM, 4 days	reversed tumor necrosis factor (TNFα)-induced proinflammatory cytokine gene expression, ↓gene expression of C/EBPα and PPARγ and protein expression of C/EBPα, PPAR γ, and FABP4	(69)
3T3-L1 preadipocyte	Eriocalyxin B (*Isodon eriocalyx*)	0.625-3.5 µM, 7 days	↓ accumulation of lipid droplets, triglycerides, and the expressions of C/EBPβ, C/EBPα, PPARγ, and FABP4 ↓mRNAs and proteins of CDK1, CDK2, Cyclin A and Cyclin B1	(70)
3T3-L1 preadipocyte	*Allium sativum* L.	20 µM, 8 days	↓expression of FABP4, PPARγ, C/EBPβ, Adipsin, and Adipoq, ↓actylation of	(71)

			α-tubulin, ↓SREBP1 ↑ATGL and HSL	
3T3-L1 mouse preadipocyte	*Artemisia princeps*	10, 50, 100 µg/ml, 8 days	↓mRNA and protein expression of PPARγ, C/EBPα, and SREBP-1c, ↓ phosphorylated p38, ERK, and JNK	(72)
3T3-L1 preadipocyte	*Clitoria ternatea*	500-1000 µg/ml, 9 days	↓expression of phospho-Akt and phospho-ERK1/2 signaling pathway, ↓expression of PPARγ, C/EBPα, FAS and ACC	(73)
3T3-L1 preadipocyte	Platyphylloside (*Betula platyphylla var. japonica*)	50 and 100 µM, 4 days	↑lipolysis related genes HSL and ATGL, ↑GLUT4 and adiponectin mRNA expression	(74)
3T3-L1 preadipocyte	*Garcinia cambogia* Extract and *Pear Pomace*	30 µg/ml(PE),60 µg/ml(GE), 8 days	↓expression of C/EBPα, PPARγ, and FAS ↑ expression of HSL	(75)
3T3-L1 Preadipocyte	*Hibiscus rosa sinensis*	25 and 50 µg/mL, 8 days	↑AMPK, ↓gene and protein expression of PPAR-γ, C/EBPα,SREBP-1c, FABP4, FAS, ↑adiponectin expression, ACC phosphorylation	(76)
3T3-L1 mouse fibroblast cell line	Do In Seung Gi-Tang (DISGT)	62.5, 125, 250 and 500 µg/ml, 7 days	↓fat droplet formation, TG accumulation, leptin production and cytokine content, ↓protein expression levels of PPAR-γ, C/EBPα	(77)
preadipocyte 3T3-L1	Cocoa tea (*Camellia ptilophylla*)	50, 100, 200 µg/ml, 8 days	↓expressions of PPARγ and C/EBPα, SREBP-1c, FAS, ACC, FAT, SCD-1, ↓JNK, ERK and p38 phosphorylation	(78)
3T3-L1 preadipocyte	*Porphyra yezoensis*	5, 10, 15 mg/ml, 9 days	↓lipid accumulation, ↓viability of preadipocytes, ↑apoptosis of mature adipocytes, ↓total glutathione Content and GSH/GSSG ratio	(79)
3T3-L1 preadipocyte	Samsoeum	25, 50, 100, 200, 400 µg/ml, 8 days	↓intracellular TG, inactivated GPDH, ↓mRNA expression of PPARγ, C/EBPα, FAS, LPL, FABP4 ↑phosphorylation of ERK1/2	(80)
3T3-L1 preadipocyte	Ivy gourd (*Coccinia grandis* L. Voigt)	50, 200, 300, 500, 800 µg/ml 8 days	↓intracellular fat accumulation, ↓expression of PPARγ, C/EBPα, adiponectin, and GLUT4	(81)
Mouse C3H10T1/2 and 3T3-L1 cell lines	Butein (*Rhus verniciﬂ ua*)	1, 5, 20, 40 µg/ml, 7 days	activate TGFβ, suppressed signal transducer and activator of STAT3	(82)
3T3-L1 adipocyte	Kaempferol (*Rhizoma Polygonati*	0.5 mg/mL of RPF extract and 40 µM of	↓expression of PPARγ, Cebpβ, SREBP1, Rxrβ, Lxrβ, Rorα and Gpd1, Agpat2, Dgat2	(83)

	falcatum)	kaempferol, 10 days	↑Tnfα, Lsr, and Cel	
3T3-L1 preadipocyte	desmethylicaritin	0.1, 1, 10 µM,<br>8 days	↑clonal expansion,<br>↓mRNA expression of PPARγ, C/EBPα, LPL and FABP4<br>↑mRNA expression of Wnt10b,<br>↑protein expression of β-catenin	(84)
3T3-L1 preadipocyte	P-synephrine (*Citrus aurantium*)	1, 10 µM, 6 days	↓expression level of C/EBPα, PPARγ and aP2, activated PKB/Akt pathway, ↓GSK3β activity	(85)
3T3-L1<br>preadipocyte	*Chrysanthemum indicum*	0.4, 1, 2 mg/ml,<br>8 days	↓accumulation of lipid droplets and mitotic clonal expansion,<br>↓activation of Akt and ERK1/2	(86)
3T3-L1 preadipocyte	S-Petasin<br>(*Petasites japonicas*)	0.31, 0.62, 1.55 µM, 8 days	↓glucose uptake,<br>↓TG accumulation,<br>↓expression of PPAR-γ	(87)
3T3-L1 adipocyte	Myricetin	10, 50, 100 µM, 48h,<br>8 days	↓mRNA and protein levels of PPARγ, C/EBPα,<br>↓mRNA levels of C/EBPβ, SREBP1-c, PGC-1, aP2, LPL, GLUT4,<br>↓phosphorylation of ERK, JNK and P38,<br>↑lipolysis in adipocytes	(88)
3T3-L1 adipocyte/<br>preadipocyte	Raspberry ketone	1, 10, 20, and 50 µM, day 2 to day 8 (preadipocyte), 24 h on day 12 (adipocyte)	↓expression of of PPARγ, C/EBPα and aP2,<br>↓mRNA levels of ACC1, FASN, SCD1,<br>↑transcriptional activities of ATGL, HSL and CPT1B	(89)
3T3-L1 preadipocyte	*Zanthoxylum schinifolium*	50, 100, 150, 200 µg/ml,<br>8 days	↓PPARγ, C/EBPα, and C /EBPβ,<br>↓ERK, PI3K/Akt pathways	(90)
3T3 L1 preadipocyte	Oroxylin A<br>(*Oroxylum indicum*)	10-40 µM,<br>8 days	↓intracellular lipid accumulation,<br>↓nuclear translocation of PPARγ,<br>↓mRNA expression of FAS and LPL,<br>↑TNF-α secretion, lipolysis<br>↓Akt phosphorylation	(91)
3T3-L1 preadipocyte	2,4,5-<br>Trimethoxybenzaldehyde	100 µg/ml<br>8 days	↓protein levels of MEK, ERK, C/EBPs, PPAR, ADD1 and ACC,<br>↑hydrolysis of TG(↓perilipin A and ↑HSL)	(92)
3T3-L1 preadipocyte	1β-hydroxy-2-oxopomolic acid<br>(*Agrimonia pilosa*)	25, 50,<br>and 100 µM,<br>8 days	↓expression of PPAR, C/EBPα, GLUT4, aP2, ADD1/SREBP1c, FAS,<br>↓expression of PPAR and C/EBPα protein levels	(93)
3T3-L1 cell line	*Spirodela polyrhiza*	20, 40, 100, 200 µm/mg,<br>9 days	↓C/EBPα and PPAR protein expression level	(94)
3T3-L1 preadipocyte	*Alnus hirsuta f. sibirica*	10, 25, 50 µm, 8 days	↓protein expression of C/EBPα and PPAR	(95)
3T3-L1 mouse embryonic fibroblast	*Aspalathus linearis*	0.5, 10 and 100 µg/ml,<br>9 days	↓mRNA expression of PPAR, PPARα,SREBF1 and FASN,<br>↓glycerol release<br>↓leptin secretion	(97)

3T3-L1 pre-adipocyte, mouse stromal mesenchymal C3H10T1/2 cell, bone marrow derived human mesenchymal stem cell (hMSCs)	Coagulin-L (*Withania coagulan*)	5, 10, 15 µM, Up to 6 days	↓expression of C/EBPα, PPAR, ↓MCE(↓phosphorylation of C/EBPβ) upregulation of Wnt/β-catenin pathway	(98)
3T3-L1 preadipocyte	*Oxycoccus quadripetalus*	2.5, 5, 10 and 20 mg/ml 8 days	↓number of adipocytes and lipid accumulation, ↑lipolysis ↓expression of PPAR, C/EBPα and SREBP1	(99)
3T3-L1 preadipocyte	Apigenin (*Daphne genkwa* Siebold et Zuccarini)	30 and 70 µM 6 days	↓ the mitotic clonal expansion ↓PPAR and C/EBPα levels upregulation of the expression of multiple C/EBPβ inhibitors	(100)
Murine 3T3-L1 preadipocyte	Kaempferol (*Nelumbo nucifera*)	2.5, 5, 10, 20, 40 µM 7 days	↓TG accumulation ↓mRNA or protein levels of C/EBPα, PPAR, RXRα, LXRα, and SREBP-1c stimulate fatty acid oxidation signaling	(101)
3T3-L1 preadipocyte	Agrimol B (*Agrimonia pilosa Ledeb.)*	10, 3 µM 6 days	↑cytoplasm-to-nucleus shuttle of SIRT1 ↓PPAR, C/EBPα, FAS, UCP-1, and apoE expression	(102)
3T3-L1 pre-adipocyte	Cis-3,4-diisovalerylkhellactone (cDIVK) (*Peucedanum japonicum*)	30 and 50 µM, 8 days	↑glucose uptake, ↑AMPK activation, ↑protein and mRNA expression of C/EBPα, PPAR and SREBP-1c	(103)
293T, HepG2, and 3T3-L1 cell	*Dendropanax morbiferus*	50, 100, 300, 500 µg/ml 7 days	↓intracellular triglyceride levels and glucose uptake, ↓ expression levels of FAS, PPAR, SREBP1, and C/EBPα	(104)
3T3-L1 preadipocyte	*Boussingaulti gracilis*	10, 50, and 100 lg/mL 6 days	↓lipid accumulation, ↓expression of PPAR, SREBP1, and C/EBPα ↑phosphorylation of AMPK	(105)
The 3T3-L1 fibroblast	Mulberry leaf ethanol extract	10, 25, 50, and 100 µg/ml 8 days	↓protein levels of PPAR, PGC-1α, FAS, and adiponectin and C/EBPα	(106)
3T3-L1 preadipocyte	*Tropaeolum majus*	20, 300, and 500 µg/mL 8 days	↓expressions of C/EBPα and SREBP1 and PPAR γ	(107)
3T3-L1 preadipocyte	*Cornus kousa*	5, 30, 60, 100 µg/ml 8 days	↓angiogenesis, ↓PPAR, CCAAT, C/EBPα, aP2, FAS, and LPL, ↑AMPK activation	(108)
3T3-L1 preadipocyte	Bilobalide (*Ginkgo biloba*)	25, 100 µM 5 days (day 4 to day 8)	↓3T3-L1 preadipocyte diferentiation and intracellular lipid accumulation, ↑phosphorylation of AMPK, ACC and HSL	(109)
3T3-L1 preadipocyte	Mesonosides A-H	20, 40 µM	↓lipid accumulation and protein levels of C/EBPα,	(110)

	(*Mesona procumbens*)	8 days	PPAR γ	
Mouse 3T3-L1 pre-adipocytes	Mangiferin (MGF) and mango leaf tea (MLT)	100 μM, 8 days	MLT: ↓TG levels, ↑adiponectin levels, ↑glucose uptake, ↑FOXO1 and ATGL, ↓ACC MGF: ↑glucose uptake, ↓ACC,	(111)
Mouse 3T3-L1 preadipocyte	Ginger (*Zingiber officinale Roscoe*)	2 μg/ml, 8 days	↓lipid content ↑glycerol concentration ↓expression of C/EBPα, FABP4 and FASN	(112)
human adipocytes	Ononin and maackiain (*Ononis spinosa* L.)	5-50 μM, 24 h	↑SIRT1 by ononin ↓ PI3K, PPARγ, C/EBPα signaling by maackiain ↓CEBPA, AKT, SREBP1, ACC and ADIPOQ by maackiain	(114)
human visceral adipose tissue (vASCs)	Resveratrol and piceatannol	5, 10, and 20 μM, 14 days	↓expression level of C/EBPα, PPARγ, aP2	(115)
human adipose-derived stem cell	Muscadine grape seed oil, tocotrienol-rich fraction	200 μM 5.7 μg/ml 10 days	↓mRNA and protein expression of PPAR and aP2 ↓LPS-induced proinflammatory gene expression, IL-6 and IL-8	(116)
primary human preadipocyte	*Momordica charantia* (bitter melon)	0.5, 1, 2%	↓mRNA expression of PPAR, SREBP1c and adipocytokine, resistin	(117)
mesenchymal stem cells from human adipose tissue	*Citrus bergamia*	10 or 100 μg/mL 14 days	↓lipid accumulation ↓PPAR-γ, A-FABP, ↑Phosphorylation of AMPKα1/2 ↑ATGL,HSL, and MGL	(118)
Human Visceral Adipose-Derived Stem Cell	Decursin (D) and Decursinol angelate (DA) (*Angelica gigas* Nakai)	40 μM 14 days	↓mRNA and protein levels of C/EBPα, PPAR, aP2, FAS and ACC, ↑activation of −β catenin signaling pathway	(119)
Human Monocytic Leukemia Cells (THP-1 cells)	*Aspalathus linearis* (Rooibos)	50 μg/mL, 8 days	↓IL-17a, INF-γ, IL-12, IL-23 secretion ↓lipid accumulation ↓gene expression of PPAR,Ap2 ↑gene expression of lipase E and HSL	(120)
